# Genome-Wide SNP-Genotyping Array to Study the Evolution of the Human Pathogen *Vibrio vulnificus* Biotype 3

**DOI:** 10.1371/journal.pone.0114576

**Published:** 2014-12-19

**Authors:** Nili Raz, Yael Danin-Poleg, Ryan B. Hayman, Yudi Bar-On, Alex Linetsky, Michael Shmoish, Eva Sanjuán, Carmen Amaro, David R. Walt, Yechezkel Kashi

**Affiliations:** 1 Faculty of Biotechnology and Food Engineering, Technion – Israel Institute of Technology, Haifa 32000, Israel; 2 Department of Chemistry, Tufts University, Medford, Massachusetts, United States of America; 3 Bioinformatics Knowledge Unit, Lorry I. Lokey Interdisciplinary Center for Life Sciences and Engineering, Technion – Israel Institute of Technology, Haifa 32000, Israel; 4 Department of Microbiology, Faculty of Biology, University of Valencia, Valencia, Spain; St. Petersburg Pasteur Institute, Russian Federation

## Abstract

*Vibrio vulnificus* is an aquatic bacterium and an important human pathogen. Strains of *V. vulnificus* are classified into three different biotypes. The newly emerged biotype 3 has been found to be clonal and restricted to Israel. In the family *Vibrionaceae*, horizontal gene transfer is the main mechanism responsible for the emergence of new pathogen groups. To better understand the evolution of the bacterium, and in particular to trace the evolution of biotype 3, we performed genome-wide SNP genotyping of 254 clinical and environmental *V. vulnificus* isolates with worldwide distribution recovered over a 30-year period, representing all phylogeny groups. A custom single-nucleotide polymorphism (SNP) array implemented on the Illumina GoldenGate platform was developed based on 570 SNPs randomly distributed throughout the genome. In general, the genotyping results divided the *V. vulnificus* species into three main phylogenetic lineages and an additional subgroup, clade B, consisting of environmental and clinical isolates from Israel. Data analysis suggested that 69% of biotype 3 SNPs are similar to SNPs from clade B, indicating that biotype 3 and clade B have a common ancestor. The rest of the biotype 3 SNPs were scattered along the biotype 3 genome, probably representing multiple chromosomal segments that may have been horizontally inserted into the clade B recipient core genome from other phylogroups or bacterial species sharing the same ecological niche. Results emphasize the continuous evolution of *V. vulnificus* and support the emergence of new pathogenic groups within this species as a recurrent phenomenon. Our findings contribute to a broader understanding of the evolution of this human pathogen.

## Introduction

*Vibrio vulnificus* is a gram-negative halophilic bacterium that is naturally found in marine and estuarine environments the world over [1]–[3]. It was first isolated in 1976 [4]. It is a highly invasive pathogen responsible for infections in both fish and humans [1], [2], [5]. Human infections are acquired through consumption of contaminated seafood or through skin wounds. The main symptoms are gastroenteritis, wound infection and septicemia, frequently leading to limb amputation and sometimes even death [2], [3]. Strains of *V. vulnificus* are classified into three biotypes based on phenotypical characteristics and differences in host range [5]–[8]. Biotype 1 clinical strains are responsible for most human infections worldwide [2], [9], whereas biotype 2 strains are primarily considered pathogens of eels, and are divided into 3 serovars, with serovar E defined as a zoonotic variant of the *V. vulnificus* species [5], [10], [11]. The most recently emerged group is biotype 3, which appears to be clonal and geographically restricted to Israel [6], [12], where it has caused an outbreak of wound infections and bacteremia among Israeli fish farmers and consumers of *Tilapia* fish. This biotype has been shown to possess biochemical properties that differ from those of biotypes 1 and 2 [6], and to include strains that are potentially more pathogenic than the biotype 1 strains present in Israel [13]. The clinical relevance of *V. vulnificus* has led to attempts to find indicators of its virulence and pathogenicity [14]–[19], as well as studies into its genetic diversity using various molecular tools, mostly aimed at differentiating clinical from environmental strains [18]–[24]. Among these tools are molecular typing methods based on a few (<20) genomic loci [12], [21], [25]–[28], including multilocus sequence typing (MLST). This latter tool was used with various collections of *V. vulnificus* isolates (composed of 63 and 115 strains), dividing the species into a few distinct evolutionary lineages with indications of different pathogenic potential [29], [30]: lineages (L) III and I contained isolates of biotype 1 and biotypes 1 and 2, respectively, whereas LII was made up of biotype 3 strains from Israel [30].

Recently, studying the genetic relationships of 188 environmental and clinical isolates based on variations at 12 variable number tandem repeat (VNTR, also termed simple-sequence repeat—SSR) loci [27], we confirmed the clonality of biotype 3, forming a genetic group distinct from biotypes 1 and 2. Furthermore, we found a new phylogroup, clade A, which includes both environmental and clinical isolates and presents biochemical characteristics that differ from those of biotypes 1 and 3 [27]. Clade A strains were isolated from the same ecological niche from which biotype 3 emerged, indicating aquaculture as a source for new emerging strains. The homogeneity of biotype 3 was also demonstrated by Bisharat et al. [26], who showed that these strains form a genetically distinct group, and suggested that this is a hybrid clone that may have evolved by genome hybridization of two different and independent populations. However, to better understand bacterial evolution and to infer the evolutionary origins of strains, variation data from multiple genetic loci are required because various genomic regions may have different origins. The recent availability of genomic sequences has allowed us to focus on comparing whole-genome data, albeit for only those few strains whose genomes have been fully sequenced [16], [31]. Nevertheless, DNA microarrays designed to represent large genome segments enable testing a large number of strains, making them a powerful high-throughput tool [32], [33]. These genotyping arrays are mostly based on single-nucleotide polymorphisms (SNPs), which are a major source of genetic variability that is fixed in the population and accumulates with time, and thus harbor the history of the specific DNA sequence [34]. SNPs have been used for genotyping and classification of isolates in various bacterial species [35]–[37] and could further serve for population studies via haplotyping [38]–[40]. Haplotype analysis enables tracing the evolutionary origin of segments in the genomes of bacterial strains. Different genome segments may have different evolutionary histories, with changes being due mainly to horizontal gene transfer (HGT) events. HGT occurs frequently in nature and plays an important role in bacterial evolution in general and in *Vibrio* in particular, contributing to the formation of new strains and species [41]–[43].

The GoldenGate SNP genotyping assay from Illumina [44], [45] is one genotyping technology that has been used in many genetic studies, including the International Human HapMap Project [46] and several genomic studies of crop species [47]–[49]. This high-throughput assay is not yet routinely used with bacteria, although it has been recently applied for SNP typing of several bacterial species, including *Salmonella* serovar Typhi [50]–[52].

In this work, we studied the evolution of the human pathogen *V. vulnificus* by high-throughput genotyping of a large and diverse panel of *V. vulnificus* isolates using genome-wide coverage. A custom SNP-genotyping microarray implemented on the Illumina GoldenGate platform was developed based on *in silico* data mining; 254 environmental and clinical *V. vulnificus* isolates were genotyped at 570 SNPs. The genotyping results were then used to infer the phylogenetic relationships between various *V. vulnificus* strains and specifically to follow the possible genomic origin of the recently emerged biotype 3.

## Materials and Methods

### Bacterial strains

A total of 254 *V. vulnificus* isolates were tested in this study (S1 Table). Source and procedure of isolation were previously described for most strains [12], [13], [23] excluding 16 strains that were isolated in this study from environmental fish samples in Israel during September-October 2008. These 16 isolates were retrieved from gills and fins/scales of Tilapia fish originated in artificial fish ponds (Kibbutz Ein HaMifratz and Kibbutz Afek, see ethics statement below) in the western Galilee region of Israel, using the previously described procedure [13].

The collection contained isolates from all three biotype groups identified in *V. vulnificus*, isolated from different geographical regions (Australia, Belgium, Denmark, France, Israel, Japan, Norway, South Korea, Spain, Sweden, Taiwan and USA) over the last 30 years (1979–2009). Most of the Israeli environmental strains were isolated between 2004 and 2009. Of the 254 isolates tested, 114 were recovered from clinical sources (wound infections and blood) and 140 from environmental sources (fish, oyster, eel, seawater, and fish-pond sediment).

All *V. vulnificus* strains were grown on thiosulfate–citrate–bile salts–sucrose (TCBS) agar and stored at −80°C in Luria Broth (LB) supplemented with 50% (vol/vol) glycerol.

High-quality DNA extraction from pure cultures was performed using a DNeasy Blood & Tissue kit (Qiagen) according to the manufacturer's instructions. DNA concentrations were quantified using the Quant-IT kit (Invitrogen).

### Ethics statement

The microbial samplings were taken from dead Tilapia fish at a local fish store in “Ein HaMifratz”, as part of the food chain while following the origin of the artificial pond where the fish were grown. No approval was required from any animal ethics committee since the tests were under the category of fresh sea food sampling, thus the fish were not scarified for the purpose of this study. The fish origin was from aquaculture fish pond of Kibbutz “Ein HaMifratz” and “Kibbutz Afek”, private land, in the western Galilee region of Israel (GPS coordinates 32.861888, 35.109086). Also we confirm that the field studies did not involve endangered or protected species.

### Construction of *V. vulnificus* SNP-variation database

Two strategies were adopted to select informative SNPs that would differentiate among a large and diverse panel of *V. vulnificus* isolates: random comparative SNP data mining and selection from targeted genes.

#### Comparative *in silico* genome-wide SNP discovery based on two biotype 1 genomes

An in-house genome-wide sequence comparison computer program was used to mine SNPs from two available *V. vulnificus* biotype 1 whole-genome sequences: YJ016 (NC_005139.1) [53] and CMCP6 (NC_004459.2) [54]. The program compares whole-genome sequences of different strains, aligns them by gene name in upstream, downstream and coding regions, outputs the allocation of SNPs and directs the selection of conserved sequences flanking each SNP to be used as probes. The program selects only sequence areas, on both sides of the SNP, which fulfill the criteria for initial probe design targeted to the GoldenGate assay (Illumina, San Diego, CA).

#### SNP discovery in targeted genes based on three genomes of biotypes 1 and 3

Twenty-four targeted genes were selected from the draft genomic sequence of biotype 3 strain VVyb1(BT3) (the provisional version at that time [55]) and compared to the genomic sequences of two *V. vulnificus* biotype 1 strains: YJ016 and CMCP6. These included 11 virulence-associated genes that were selected from the Virulence Factors of Bacterial Pathogens database (VFDB) (http://www.mgc.ac.cn/VFs/) and 13 plasmid and unique biotype 3 genes (S2 Table). All comparisons and testing of uniqueness were carried out using NCBI BLAST against microbial genomes (http://www.ncbi.nlm.nih.gov/sutils/genom_table.cgi) and The RAST Server http://rast.nmpdr.org/ (Aziz et al., 2008), followed by multiple alignment using ClustalW (ftp://ftp-igbmc.u-strasbg.fr/pub/ClustalW/). Sequences were then converted to Excel using an in-house ‘convert to Excel’ program followed by SNP selection from the aligned sequences.

#### SNP discovery using sequence analysis of targeted genes in 30 strains

Twenty candidate genes were selected and sequenced on a subset of 30 isolates that included strains of the three biotypes as well as the clade A group, from clinical and environmental sources. The genes included seven previously studied housekeeping genes (*glp, gyrB*, *mdh*, *lysA, pntA, pyrC* and *tnaA*) [26], five conserved hypothetical genes (VV0048, VV0178, VV0415, VV1197 and VV1483) [27], the 16S rRNA gene [56], and seven newly selected genes (S3 Table). PCRs, sequencing and result analyses of selected loci were performed as previously described [27]. SNPs were selected from multiple sequence alignment of the genes.

The newly selected genes were tested for uniqueness throughout the *V. vulnificus* genomes using BLAST as described above. PCR primer design was based on either the sequences of *V. vulnificus* YJ016 [53] or the newly sequenced biotype 3 strain, VVyb1(BT3) [55] (S3 Table), using Gene Runner software (Version 3.05, Hastings Software Inc.).

### SNP custom array

A custom Illumina GoldenGate assay was designed based on the SNP-variation database. Sequences surrounding each known SNP were submitted to Illumina for validation for the GoldenGate platform—the selected SNP locus was required to have at least 60 bp of unvarying flanking sequence on each side of the SNP to allow sufficient room for the probe to bind. A total of 1,071 SNPs were validated (final score >0.6 and without error codes), of which the top-ranked 574 were selected for genotyping. Thirty-one of these SNPs, whose sequences were also determined by sequencing in a panel of 30 strains included in the array (see above), served for array quality control. The full list of SNP loci and alleles used in the analysis is given in S4 Table.

### *V. vulnificus* SNP genotyping

The custom Illumina GoldenGate assay was used to genotype 254 *V. vulnificus* isolates [57]. Samples (250 ng) were genotyped on a Sentrix Universal-16 BeadChip array following the Illumina protocols [44] and assayed using the custom oligo pools. These DNA samples were ranked according to p10GC score [58] after genotyping, giving an average of 0.55 which indicates good quality DNA. DNA samples, tested in triplicate, were arrayed randomly in a 96-well plate, along with control samples in each plate.

A total of 46 BeadChip arrays were utilized and scanned with the Illumina BeadStation 500G. The GoldenGate genotyping assay is based on a highly multiplexed, allele-discriminating, extension reaction [44]. Briefly, two allele-specific oligonucleotides and a locus-specific oligonucleotide are used to query each SNP. The Illumina GoldenGate assay generates a detection signal for each of the two target alleles at each SNP locus, in each sample. These hybridization intensities are typically normalized and converted to genotype calls, by GenomeStudio software which yields three distinct genotype clusters consisting of the two homozygous alleles (alleles 1 and 2) and one heterozygous genotype cluster. This general analysis is mostly aimed at calling genotypes in human and other diploid organisms. Since *V. vulnificus* is haploid, only homozygous clusters are expected.

Thus here, samples with no signal for both alleles are assigned to a third allele. To fit the ‘no signal’ cluster in place of the heterozygous cluster, signals from the GoldenGate assay were analyzed using Illuminus-P as previously described [50], [59] and a newly developed algorithm was used, Calc Allele, that fits the ‘no signal’ cluster in place of the heterozygous cluster and determines the allele call for each sample. Then an additional developed algorithm (using the R platform) was used, Calc call, that determines the consistency between replications and generates the final genotype call for each strain The described procedure was first tested on SNPs of the array quality control panel. A third algorithm was developed to compare the three genotype-calling algorithms. This algorithm compares the genotyping results from the GoldenGate array to those obtained by standard sequencing (array quality control), gives a concordance score for each SNP locus and the sum of concordances for each tested algorithm.

### Phylogenetic analysis based on GoldenGate and MLST data

Two phylogenetic analyses were performed for the array data: parametric and nonparametric.

#### Parametric analysis—based on sequence data

The array data of the 254 strains at 570 SNPs were concatenated to give a specific sequence for each isolate. The same was applied to the MLST data for a panel of strains at 20 specific genes, and multiple alignment of the sequences was performed using SeqScape software (Applied Biosystems). The output files of both the array and MLST (FASTA format) were converted to MEGA format, and a phylogenetic tree was constructed with MEGA 5 [60] by using the maximum-likelihood method and Kimura two-parameter distance for all substitutions; gaps were treated as missing data using the “pairwise deletion” option, and the inferred phylogenies were tested with 1,000 bootstrap replications.

#### Nonparametric analysis—based on allelic variation

A nonparametric analysis of allelic variation was used for phylogenetic analysis of the array data from the SNP alleles. The data for all genotypes were scored as present (1) or absent (0) for each of the alleles (1, 2 and null alleles) at a specific SNP locus. Genetic relationships among strains were inferred from the GoldenGate data using Jaccard similarity coefficient, and the distance matrix was generated with PAST software Version 1.94b [61]. A phylogenetic tree was constructed with MEGA 5 [60] by using the minimum-evolution method. Similarly, a nonparametric analysis of allelic variation was used for array quality control by calculating the Nei coefficient of association and generating the corresponding matrix with SAS 8.02, followed by minimum-evolution cluster analysis using MEGA 5.

Variation among *V. vulnificus* biogroups and phylogroups was analyzed by PowerMarker V3.25 [62].

### Haplotype analysis

A heat map of the 570 SNPs was calculated by using the ‘heat map’ module of the statistical software environment R, version 2.14 to generate cluster analysis of 68 biotype 3 isolates and 59 representative biotype 1 isolates. Biotype 1 strain selection was based on the parametric phylogenetic tree. 40 unique biotype 3 SNPs were excluded from all further analyses (as these were present only in biotype 3 isolates), leaving 530 SNPs as the full genotyping dataset (composed of datasets of 411 and 119 SNPs from chromosomes 1 and 2, respectively).

The GoldenGate genotyping datasets of biotype 1 isolates were processed in relation to a biotype 3 synthetic haplotype. The original SNP-genotyping data was transformed so that instead of each of the alleles, a value that represents whether that allele is similar to the parallel allele in the biotype 3 synthetic haplotype (1 for similar, 0 for not similar) was placed. In total, three matrices were constructed: for each chromosome separately and for both chromosomes together.

#### Grouping of relative biotype 3 datasets

Principal component analysis (PCA) [63], [64] was performed on the resulting matrices to test clustering of biotype 1 isolates relative to biotype 3, using PAST software version 2.06 [61]. PCA is a procedure for finding hypothetical variables (components) that account for as much of the variance in the data as possible. Analyses for each chromosome separately did not enhance result accuracy, where chromosome 1 separately gave results similar to those for both chromosomes together, and therefore PCA results of each chromosome separately are not presented.

#### Construction of group origin map

The biotype 3 synthetic haplotype was marked according to the three origin groups revealed by the PCA analysis. The occurrence of each allele was counted in each of the three biotype 1 groups. The origin of the allele of biotype 3 was determined based on the relatively higher appearance of that allele in one of the three biotype 1 groups (while normalizing for group size), resulting in a group origin map using the developed algorithm (written in Perl and Excel).

## Results

### Development of a custom microarray

A high-throughput custom SNP-genotyping microarray was developed to study the evolution of the human pathogen *V. vulnificus* in a large and diverse panel of *V. vulnificus* isolates. The first step was construction of a SNP-variation database using two approaches for SNP selection: random comparative SNP data mining and selection from targeted genes. 472 SNPs were selected by *in silico* genome-wide comparison of genes from two *V. vulnificus* biotype 1 genomes (YJ016 and CMCP6) [53], [54] and one SNP per gene was picked based on the findings that SNPs are randomly distributed along the two chromosomes (S1 Fig.), ensuring that the genes can be randomly selected for the construction of the variation database. By comparing sequences from the three genomes of biotypes 1 (YJ016 and CMCP6) and biotype 3, 49 SNPs were selected from 24 targeted genes including virulence, plasmid and biotype 3-unique genes originating from the draft genome of biotype 3 [55]. To identify SNPs from a larger and more diverse panel of strains, a sequence analysis, MLST of 20 candidate genes was carried out for representative isolates from biotype 1, 2, 3 and clade A groups, resulting in the identification and selection of 53 SNPs. This approach was applied to targeted genes such as conserved hypothetical genes, the 16S rRNA gene, housekeeping genes and newly identified genes; 31 SNPs originating from 12 genes served for array quality control (see further on). The final custom array contained 574 SNPs from 516 genes covering ∼10% of the genes in the *V. vulnificus* genome.

### Genome-wide genotyping using the developed array and array quality control

The designed custom Illumina GoldenGate array was used to genotype 254 clinical and environmental *V. vulnificus* isolates representing the three biotypes and phylogroup clade A recovered over a 30-year period from a wide variety of hosts and habitats all over the world. A unique pattern was achieved for every strain based on the response for each SNP locus interrogated. Genotyping revealed one of the two alleles for each strain based on the designed probe (S2 Fig.), while strains that did not contain either allele were not expected to show any signal in the assay, and were thus designated ‘no product’. Since the GoldenGate assay is generally used for genotyping diploid organisms, and in order to fit the ‘no signal’ cluster for this haploid bacterial panel in place of a heterozygous cluster, an analytical procedure was conducted in three steps on partial array data for array quality control. The array quality control included 88 DNA samples, representing replicates of 30 *V. vulnificus* strains from the three biotypic groups and clade A, at 31 SNP loci. In the first step, three genotype-calling algorithms were tested to choose the most suitable analytical method for converting signals into alleles. The second step was determining consistency between replicates and generating the final genotype call for each strain. In the third step, GoldenGate genotyping data as obtained by each of the three calling algorithms were compared to the parallel sequencing data of SNPs.

Analysis of array quality control revealed full concordance between the replicated samples, i.e., the same genotype was observed over the replicates, yielding a reproducibility rate of 100%, and illustrating the high reproducibility of the array genotyping results. Comparison of the results obtained by the three algorithms revealed that Illuminus-P gives the best allele calls with an average (for all SNPs) concordance score of 29.2 (compatibility of 94% of the SNPs). Concordance scores of 28.7 and 28.9 were obtained with Illumina GenomeStudio software and Calc Allele, respectively (compatibility of 92.5% and 93.22% of the SNPs, respectively). Thus Illuminus-P was used throughout the study to analyze the array data.

**Figure 1:**
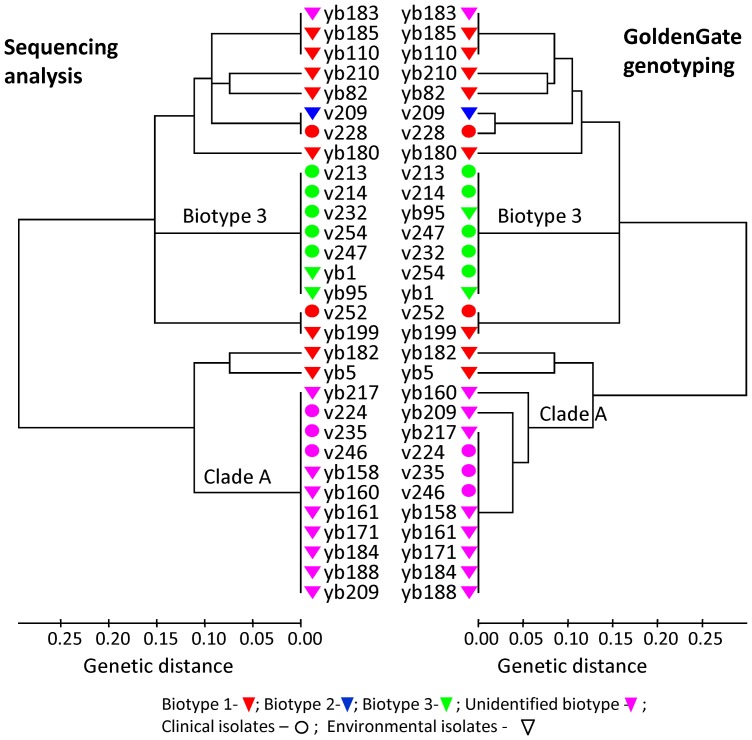
Phylogenetic relationships among 30 *V. vulnificus* strains inferred from GoldenGate genotyping results compared to sequencing. Results were based on Illuminus-P genotype calls obtained by the GoldenGate assay at 31 SNPs compared to sequencing analysis of the same 12 intergenic loci analyzed as sequence types. These loci served for array quality control. A nonparametric analysis of allelic variation was used to calculate the Nei coefficient of association and to generate the corresponding matrix with SAS 8.02, followed by minimum-evolution (ME) cluster analysis using MEGA5.

Genetic relationships among the strains inferred from the quality control data (Fig. 1) showed clustering similar to the parallel sequence data of SNPs, suggesting agreement between the array and the real sequences. Four SNPs gave inconsistent genotyping results, presenting variation between some of the strains that exhibited identical sequences (Fig. 1, e.g., clade A strains). Although only minor differences were observed between the dendrograms, these four SNPs were excluded from the final analysis. These results indicated that ∼90% of the designed SNPs had been successfully picked, supporting the use of the GoldenGate assay for phylogenetic analysis of *V. vulnificus*. The final genotyping results were obtained for each of the 254 *V. vulnificus* strains at 570 SNP loci, determined according to three replications, using the Illuminus-P calling algorithm.

### Phylogenetic study of *V. vulnificus*

The array results matrix was used to infer phylogenetic relationships among all studied *V. vulnificus* isolates. Two approaches were used to analyze the SNP data: parametric and nonparametric.

**Figure 2:**
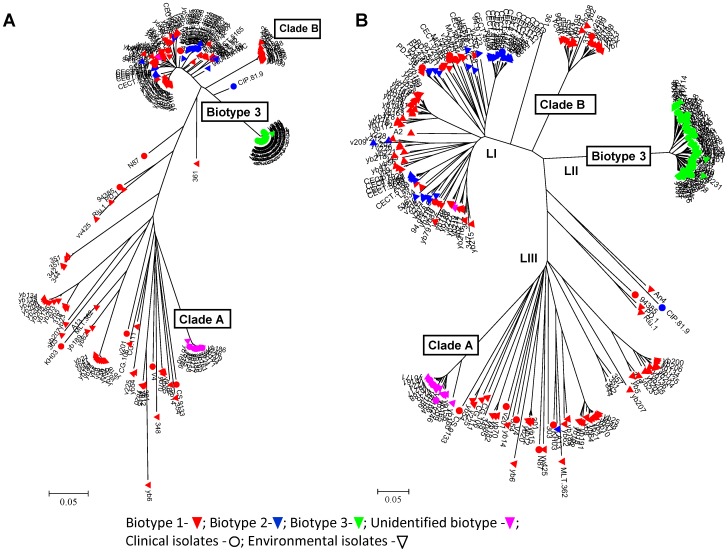
Phylogenetic relationships among 254 *V. vulnificus* isolates based on variation data at 570 SNP loci. (A) Parametric analysis: the evolutionary history was inferred using the neighbor-joining method and the evolutionary distances were computed using the Kimura two-parameter method with bootstrap analysis of 1,000 replicates. (B) Nonparametric analysis: genetic relationships among strains were inferred using the Jaccard similarity coefficient, and the distance matrix was generated with PAST software (version 1.94b). The phylogenetic tree was constructed by using the minimum evolution (ME) cluster analysis.

**Figure 3:**
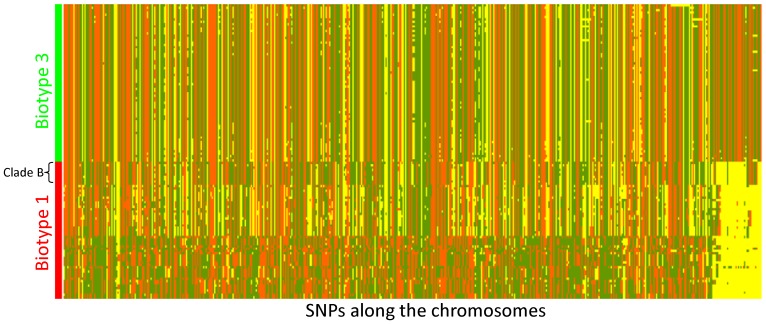
Heat map of genotyping data at 570 SNPs of 127 strains isolated in Israel. *V. vulnificus* strains (vertical axis) are arranged according to their phylogenetic relationships and marked by biogroups (68 biotype 3 isolates and 59 representative biotype 1 isolates). Allelic variations of SNPs (horizontal axis) are ordered according to their position along the two bacterial chromosomes (according to the YJ106 genome). The last 40 SNPs (on the right) were selected as unique to biotype 3 based on its draft genome (VVyb1(BT3)). Different colors represent the different alleles: allele 1 – orange, allele 2 – green, and “no product” allele – yellow.

The resulting dendrograms (Fig. 2) gave similar results, supporting the obtained clustering and strengthening the use of this SNP-genotyping array for *V. vulnificus* phylogenetic studies. Both dendrograms showed that isolates of biotype 3 form a separate, clear cluster, whereas biotype 1 was highly diverse and dispersed throughout the dendrogram. Biotype 2 was heterogenic as well and clustered into subgroups according to serovars. The dendrogram showed grouping into three main clusters, which were assigned as the three main evolutionary lineages of *V. vulnificus* [29], [30] based on the array genotyping results of previously characterized isolates belonging to the different lineages (S1 Table, [30]. Isolates of biotype 1 were split into two lineages (I and III); biotype 2 was present in LI; biotype 3 strains were exclusively present in LII, and clade A isolates were included in LIII, forming a clonal group. An additional distinct cluster (clade B) composed of Israeli environmental and clinical biotype 1 strains appeared close to LI. In general, more of the isolates recovered from environmental sources were present in LI than in LIII (107 and 59, respectively). Isolates recovered from clinical sources were present almost equally in LI and LIII (12 and 7, respectively). Biotype 2 isolates branched in LI with further subdivisions into serovars A, E, and I [30], and serovar E isolates clustered together (Fig. 3B; blue clade in LI comprising of 16 strains). Quantifying the variation among *V. vulnificus* strains (Table 1) showed very high variation (>90% diversity) within biotype 1, and rather high variation (∼60% diversity) within biotype 2. High similarity was seen within biotype 3 (20% diversity), clade A (10% diversity) and clade B (15% diversity) groups, supporting their clonality. A comparison of the array variation to that found by the MLST of 15 genes [27] revealed a certain amount of variation in our larger genotyping study for the clade A and biotype 3 groups but no evident variation when testing a few loci by MLST, indicating the contribution of the genome-wide array data to variation analysis among strains.

**Table 1:** Variation within *V. vulnificus* groups with worldwide distribution isolated between 1979 and 2009 (254 isolates).

	Total strains	Average allele frequency	Median allele frequency	Similarity
**Biotype 1**	115	0.39	0.33	8.77
**Biotype 2**	39	0.60	0.82	41.23
**Biotype 3**	68	0.82	1.00	78.07
**Clade A**	14	0.90	1.00	89.47
**Clade B**	18	0.87	1.00	85.26

### Genetic origin of the biotype 3 haplotype

**Figure 4:**
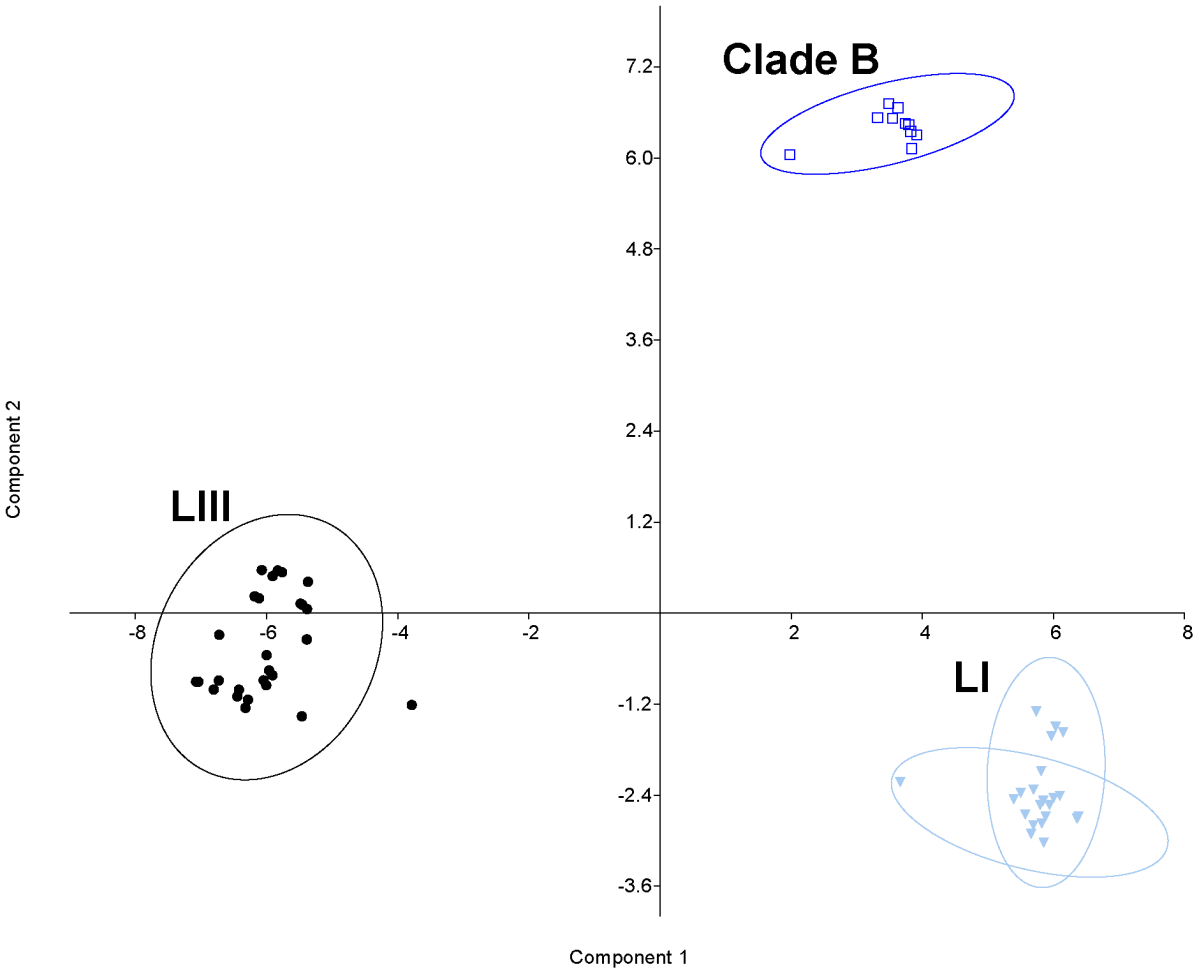
PCA of genotyping data of Israeli biotype_1 strains processed in relation to synthetic biotype_3 haplotype. Principal component analysis (PCA) was carried out on datasets containing 530 SNPs. The scatter diagram of a cluster analysis performed with PCA is shown; the strains (dots) are classified into two or three groups with the following color code: LI – light blue, azure, LIII – black, LI-clade-B – dark blue. Together, components PC1 and PC2 explain 50% of the variance (38 and 12%, respectively); 95% confidence ellipses around each group are indicated.

To understand the genome origin of biotype 3, a haplotype analysis was performed, based on the genome-wide array data, focusing on the Israeli isolates of biotype 1 that were isolated in the environmental niche from which biotype 3 emerged. As a first step, 59 representative biotype 1 strains were selected by removing isolates with a common clonal origin based on the phylogenetic dendrogram. A heat map was generated based on the 59 biotype 1 strains and all 68 biotype 3 strains, to combine the matrix of allelic variations of SNPs along the bacterial chromosomes with the phylogenetic analysis (Fig. 3). Such a presentation enables following the distribution of one or a group of SNPs (chromosome segments) according to the phylogenetic relationships of the strains (marked by biotypes). This map (Fig. 3), as well as one that included all Israeli isolates (S3 Fig.), both presented a haplotype for each strain, emphasizing the high discrimination power of the array and its ability to identify a specific isolate. Zooming out, several clear clusters were observed, as all biotype 3 isolates formed one conserved cluster alongside the widely diverse biotype 1 isolates, which formed a few clusters. Thus, to trace the evolution of biotype 3, we chose to follow the contribution of different main biotype 1 groups, rather than specific isolates, to the creation of biotype 3's genome. The concept was to process allelic-variation data from biotype 1 isolates in relation to biotype 3. Therefore, a synthetic haplotype representing biotype 3 was constructed based on the high similarity (Table 1) among biotype 3 isolates by using the most frequent allele for each of the SNPs in the array, excluding 40 SNPs selected from 17 loci (S2 Table and S3 Table) unique to the biotype 3 genome. Array results revealed that indeed these loci present a ‘no-product’ allele in most biotype 1 strains (Fig. 3, far-right SNPs in the heat map). To reveal the possible contribution of main biotype 1 isolate groups to the creation of biotype 3's genome, a matrix of the 530 SNPs' data variation relative to biotype 3 was subjected to grouping analysis. According to the heat map and the phylogenetic tree (Figs. 2, 3), we hypothesized that biotype 1 isolates could be divided into three groups: LI, LIII and clade B. Grouping by PCA strongly confirmed this hypothesis, as shown by the 95% confidence ellipses (enclosing data points belonging to a group, with the first two components, PC1 and PC2, together explaining 50% of the variance; Fig. 4). Results suggested that biotype 3's genome originates from the genomes of three main biotype 1 groups: LI, LIII and clade B.

**Figure 5:**
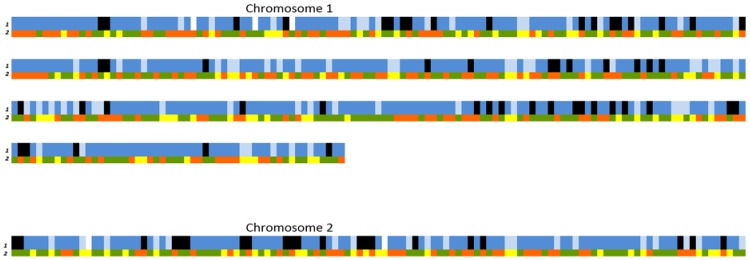
The group origin map of the biotype 3 haplotype in comparison to the synthetic haplotype. Data are arranged in three rows representing the 530 SNPs along the *V. vulnificus* chromosomes (genome). (1) The group origin map based on PCA results (Fig. 4). Blue – SNPs originated in LI-clade-B, light blue – SNPs originated in LI, black – SNPs originated in LIII; SNPs that were not found in any of the 59 tested biotype 1 strains were not colored. (2) The synthetic haplotype of biotype 3. The different alleles are color coded as follows: allele 1 – orange, allele 2 – green, and “no product” allele – yellow.

The next step was to identify the contribution of each of the groups to creation of the biotype 3 genome. A group origin SNP map for the biotype 3 genome was constructed (Fig. 5), where each of the 530 SNPs was colored according to its similarity to the most abundant alleles in each of the three main biotype 1 groups. Of these biotype 3 SNPs, 69% were similar to clade B, whereas only 16% and 14% appeared to be similar to the rest of the LI and LIII groups, respectively. The SNPs from clade B appeared in large blocks, whereas those from the other two groups were randomly distributed with no more than three adjacent SNPs.

In addition, looking at the group origin of SNPs from seven housekeeping and five conserved hypothetical genes in the synthetic biotype 3 haplotype (S4 Table), we found that their majority (52%) was similar to clade B, whereas only 16% and 13% appeared to be similar to the rest of LI and LIII, respectively, similar to the grouping of the total analyzed SNPs. Results support clade B phylogoup as the main genome origin of biotype 3.

## Discussion

A custom genome-wide SNP microarray was developed to study the evolution of the human pathogen *V. vulnificus* and to find a common ancestor between biotypes 1 and 3. Microarrays can be used to simultaneously compare multiple strains at the whole-genome level and may provide insight into the bacterial evolution [41], [42] that contributes to the emergence of new strains, such as *V. vulnificus* biotype 3. One of the challenges in array development is SNP discovery [34], [65]. Here, SNP selection was achieved using two approaches: genome-wide discovery and selection from candidate genes. Most (82%) of the SNPs were selected based on *in silico* variation between the two available *V. vulnificus* biotype 1 genomes [53], [54], enabling simple selection of multiple SNPs with high genome coverage. However, a comparison of two genomes may not represent the high variation existing in the species, such as strain-specific variation and differences between groups.To cover wider genetic variation, additional SNPs were selected from candidate genes originating from a draft biotype 3 genome [53]–[55] and from sequencing of several genes in a diverse panel of strains representing the various biogroups. Some of the targeted genes had been previously used for phylogenetic studies of *V. vulnificus* by MLST [23], [26], [27], [30], a method that has been widely adopted for the study of bacterial population structure [66]. However, this method covers only a small portion of the genome. In contrast, the final custom array implemented on the Illumina GoldenGate platform contained 574 SNPs covering ∼10% of the genes in the *V. vulnificus* genome. This array provides greater discriminatory power than any previous study of *V. vulnificus* reported to date. As such, the array allowed the discrimination of distinct strains within this species, including those belonging to clonal groups.

### Array quality control

Array quality control was based on comparing genotyping information obtained from the array technology for a small strain panel to their sequencing results. The comparison revealed high concordance between the two methods. Genetic relationships among strains inferred by the GoldenGate quality control data were in agreement with those inferred using the parallel SNP data based on the sequencing approach (Fig. 1), and also coincided with previous studies based on MLST of the same genetic loci [26], [27], [38], [67]. Results indicated that ∼90% of the SNPs included in the whole array had been chosen successfully. In addition, full concordance between the replicated samples of the quality control strains was achieved, illustrating the reproducibility of the genotyping array. Array quality control results supported the use of GoldenGate data for phylogenetic and evolutionary analysis of *V. vulnificus*.

### Phylogenetic study of *V. vulnificus*

A wide variety of strains (254) representing the three biotypes and the phylogroup clade A were successfully genotyped by the array, yielding a large allele dataset consisting of a unique SNP pattern for every strain at 570 loci. The quality of the genotyping was demonstrated by the similarity of the three replicated samples for each strain. Variation and phylogenetic analysis of GoldenGate data demonstrated the high genetic diversity among *V. vulnificus* isolates, mainly those of biotypes 1 and 2, compared to the high similarity present within biotype 3 and clade A groups, supporting previous works [26], [27]. Isolates of biotype 3 and clade A formed distinct and separate clusters that included both clinical and environmental strains, indicating the clonality of each of these clusters. Previously, variation within these two groups had only been shown using SSR markers that presented a relatively high mutation rate, while no sequence variation was found among isolates of biotype 3 [12], [26], [27], [67], or among isolates belonging to clade A using MLST of a few genes [27]. In comparison, significant but low variation (Table 1) was found in the extensive SNP-array study within these two groups. These results highlight the value of the genome-wide array data, and emphasize the continuous evolution of this highly pathogenic *V. vulnificus* species.

The phylogenetic tree based on our relatively large-scale genomic data showed a general subdivision of the species into three main evolutionary lineages, supporting previous studies conducted on a small number of loci [29], [30]. Both LI and LIII had a star-shaped phylogeny, as noted previously [29]. LI consisted of all biotype 2 isolates, with an internal division into main serovar groups A, E, and I, compatible with previous studies [30]. Results also confirmed that biotype 2 is polyphyletic and is only defined by the possession of a virulence plasmid that can be acquired via HGT by different clones belonging to different clades or lineages [68], [69]. Serovar E isolates associated with human vibriosis [10] clustered together, showing high similarity among strains. Interestingly, in the current study, an additional distinct phylogroup composed of Israeli environmental and clinical biotype 1 strains, clade B, appeared close to LI.

### Insight into the origin of biotype 3 via haplotype analysis

While the phylogenetic analysis revealed clustering of the various tested *V. vulnificus* isolates, providing a broad picture of their genetic distribution, haplotype SNP analysis of the array data enabled tracing the evolutionary origin of various genomic segments [38] in the newly emerged biotype 3. This highly virulent biotype, which is geographically restricted to Israel and forms a genetically distinct and homogeneous group, was proposed to have evolved from two distinct and independent biotype 1 populations in a relatively recent genome-hybridization event [25], [26], [67]. More recently, a genome sequence of a biotype 3 strain (VVyb1) showed ∼90% similarity to biotype 1 genomes [55], [70]. Taken together with the concept that biotype 1 is the most ancient and diverse biogroup (Hollis et al., 1976), we hypothesized that the donor genome that evolved into biotype 3 came from biotype 1 and apparently evolved in the ‘melting pot’ where biotype 3 was isolated, i.e. aquaculture fish farms in Israel.

In an attempt to trace the evolution of biotype 3, the alleles of a synthetic haplotype that represents this highly conserved biotype were compared with those of the Israeli biotype 1 strains. The analyzed Israeli biotype 1 isolates clustered into three different groups in relation to biotype 3—LI, LIII and clade B—implying three possible origins for the biotype 3 haplotype. Construction of a group-origin SNP map (Fig. 5) for the biotype 3 genome based on SNP similarity to these three groups revealed similarity of most of the biotype 3 haplotype to clade B, suggesting this group as a main possible origin for the biotype 3 genome. This finding was consistent with the phylogenetic tree (Fig. 2), as well as the heat map (Fig. 3), where this subgroup appeared to be closest to the biotype 3 branch. The map also showed the distribution of SNPs from various origins. The SNPs originating from clade B appeared in large blocks, whereas the SNPs similar to the two other origins (LIII and LI) were randomly distributed, mostly as single SNPs. This observation suggests that biotype 3 genome creation was based on one core genome of biotype 1, most likely belonging to phylogroup clade B, which probably acquired small chromosomal segments via HGT from other *V. vulnificus* strains and/or other bacterial species in its natural environment, such as *Shewanella* [70]. Furthermore, strains of clade B were isolated from the same environmental habitats as biotype 3 isolates, and have been identified in infected patients, showing pathogenic potential similar to that of biotype 3 strains. In addition, we previously showed that a clade B representative strain carries ‘unique’ biotype 3 genes, supporting their genomic relatedness [70]. We therefore conclude that biotype 3 and clade B have a common ancestor and that the evolution of biotype 1 was a stepwise process that depended mainly on gene transfer within species and from other bacterial species sharing the same environment, thus leading to the constant creation of new strains. We hypothesize that a single episode of genome hybridization of two bacterial populations, as suggested previously [26], might occur in *V. vulnificus*, but this is less likely to have been the main event for biotype 3 creation. The contribution of the different biotypes and their subgroups to creation of the biotype 3 genome could be more precisely determined by extensive full-genome comparison of multiple strains representing the various biogroups.

In conclusion, we describe here the first genome-wide SNP-based typing study of *V. vulnificus* strains that includes a large panel of isolates. Aside from the high discrimination power presented by the array, the phylogenetic tree inferred by the SNP-genotyping data showed formation of the main known evolutionary lineages (LI, LII and LIII) with an additional cluster, clade B, composed of Israeli environmental and clinical biotype 1 strains. Attempts to follow the evolutionary origin of the newly emerged biotype 3 indicate that its genome may have been created in an as-yet undiscovered event, based on the core genome of a biotype 1 strain most likely belonging to clade B that gained a rather small number of genes by HGT from its natural environment, leading to a change in biotype. Our results emphasize the continuous evolution of *V. vulnificus* and support the recent emergence of new pathogenic groups within this species as a recurrent phenomenon. Use of the high-throughput SNP-genotyping array enabled the study of a large panel of strains and contributes to our broader understanding of the evolution of this human pathogen.

## Supporting Information

S1 Fig **Distribution of SNPs found between CMCP6 and YJ016 genomes presented along the** ***V. vulnificus*** **YJ016 genome.**
(TIFF)

S2 Fig **Allelic variation among 30** ***V. vulnificus*** **strains at SNP locus VV1197_322.**
(TIFF)

S3 Fig **Heat map generated for the GoldenGate data at 570 SNPs distributed along the bacterial chromosomes of 185** ***V. vulnificus*** **strains isolated in Israel between 1996 and 2009.**
(TIF)

S1 Table **The** ***V. vulnificus*** **strains used in this study.**
(DOCX)

S2 Table **Targeted genes used to mine SNPs in biotype 3 and biotype 1 genomes.**
(DOCX)

S3 Table **PCR primers for sequence analysis of 30 strains performed for SNP discovery.**
(DOCX)

S4 Table **The 574 SNP loci and alleles used in this study.**
(DOCX)
