# Supplementary figures and images for: Genome-Wide SNP-Genotyping Array to Study the Evolution of the Human Pathogen Vibrio vulnificus Biotype 3
Source: PLoS One. 2014 Dec 19;9(12):e114576. doi: 10.1371/journal.pone.0114576 (PMC4272304; doi:10.1371/journal.pone.0114576)

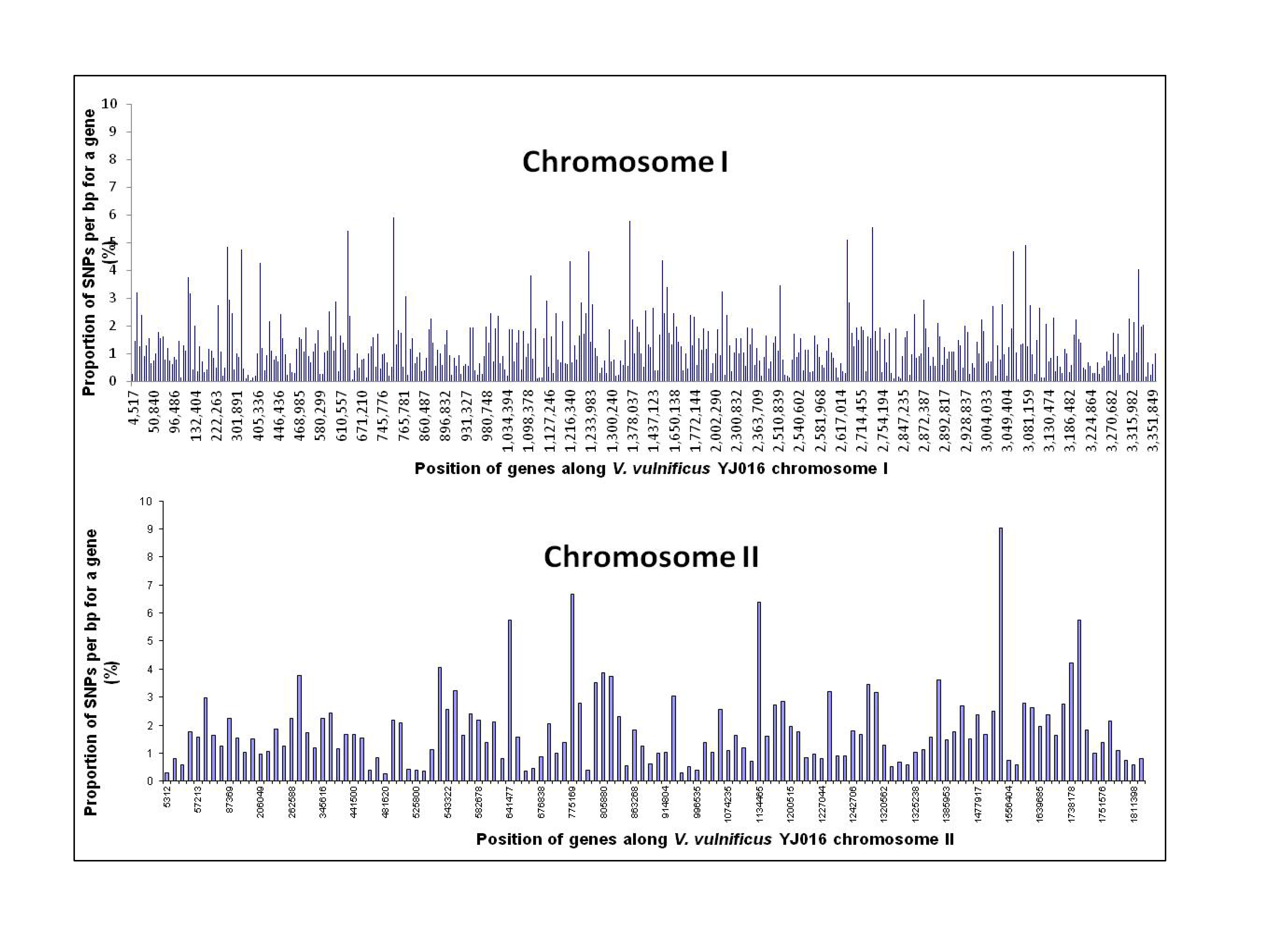

Supplement: S1 Fig — Distribution of SNPs found between CMCP6 and YJ016 genomes presented along the V. vulnificus YJ016 genome. (TIFF) [file pone.0114576.s001.tiff]

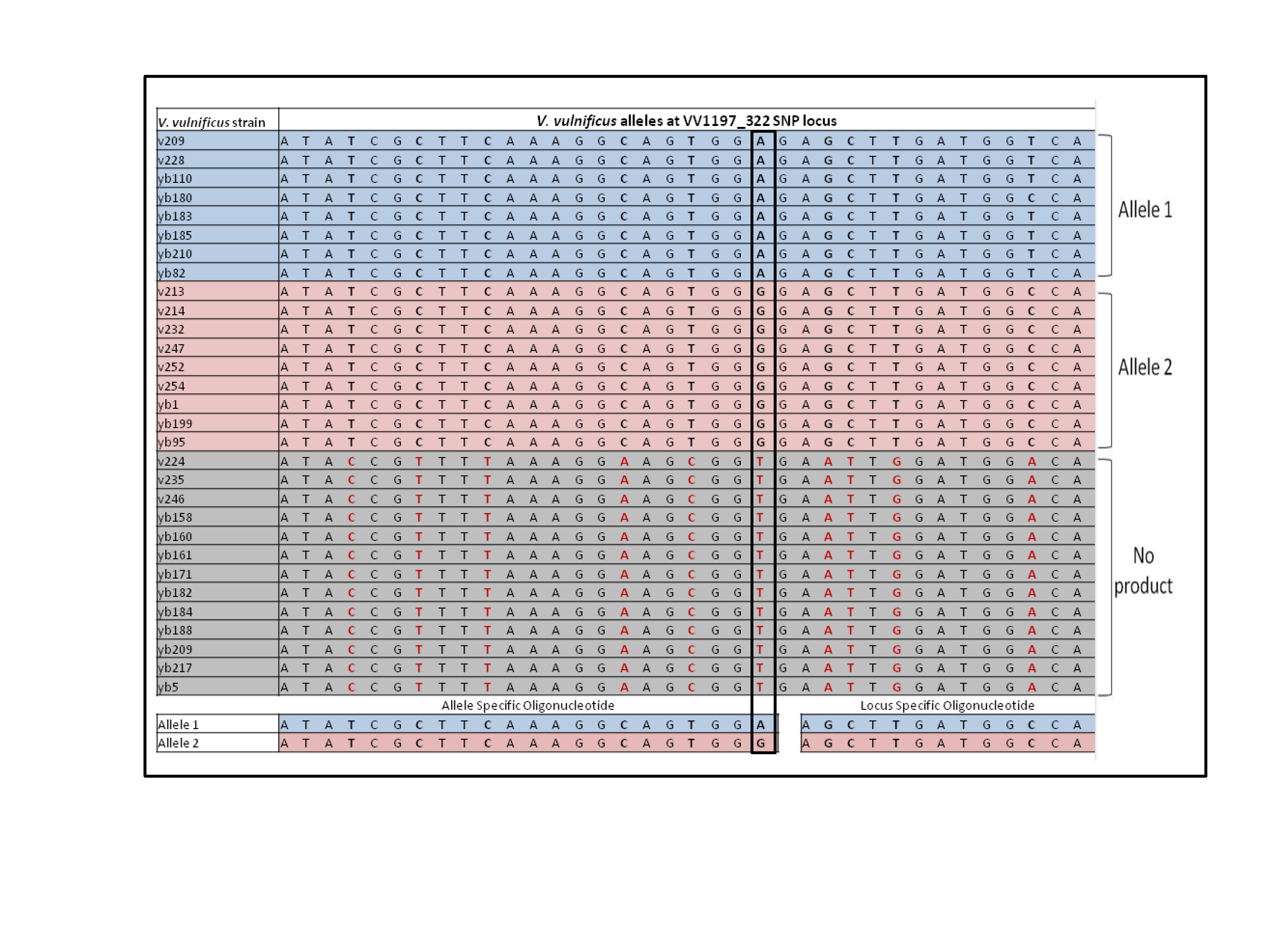

Supplement: S2 Fig — Allelic variation among 30 V. vulnificus strains at SNP locus VV1197_322. (TIFF) [file pone.0114576.s002.tiff]

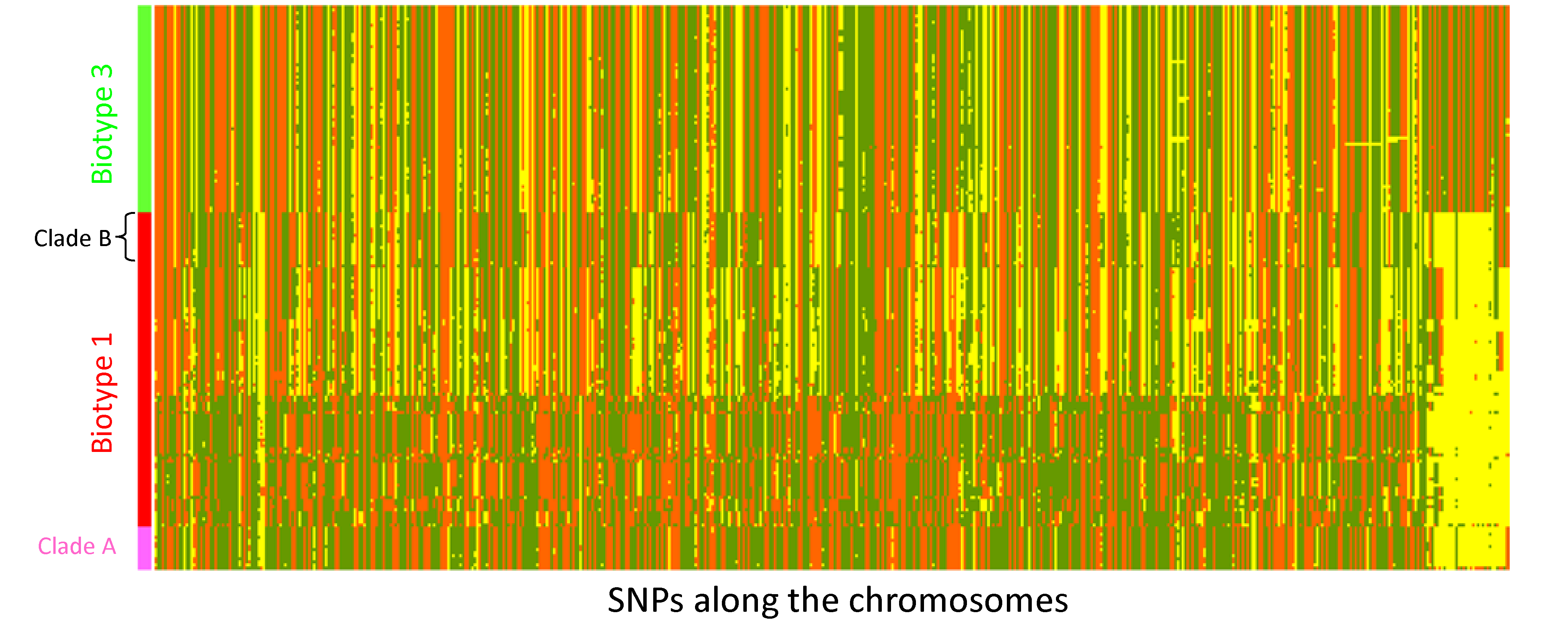

Supplement: S3 Fig — Heat map generated for the GoldenGate data at 570 SNPs distributed along the bacterial chromosomes of 185 V. vulnificus strains isolated in Israel between 1996 and 2009. (TIF) [file pone.0114576.s003.tif]
