# Supplementary material for: Genome-Wide SNP-Genotyping Array to Study the Evolution of the Human Pathogen Vibrio vulnificus Biotype 3
Source: PLoS One. 2014 Dec 19;9(12):e114576. doi: 10.1371/journal.pone.0114576 (PMC4272304; doi:10.1371/journal.pone.0114576)
Supplement: S1 Table — The V. vulnificus strains used in this study. (DOCX) [file pone.0114576.s004.docx]

Table S1*:* The *V. vulnificus* strains used in this study.

| **Strain^1^** | **Biotype^3^** | **Isolation Source** | **Country of isolation^4^** | **Year of isolation** |
| --- | --- | --- | --- | --- |
| V4 | 1 | Clinical | Australia | Unknown |
| CECT 4869 | 1 | Diseased eel | Belgium | 1990 |
| v201 (9028A/95) | 1 | Clinical | CDC^4^ | 1995 |
| v203 (9032/95) | 1 | Clinical | CDC^4^ | 1995 |
| v204 (9020/96) | 1 | Clinical | CDC^4^ | 1996 |
| v205 (9021/96) | 1 | Clinical | CDC^4^ | 1996 |
| v206 (9022/96) | 1 | Clinical | CDC^4^ | 1996 |
| 94-9-119 | 1 | Clinical | Denmark | 1994 |
| v252 (7/04) | 1 | Clinical | Israel^4^ | 2004 |
| v255 (1/09) | 1 | Clinical | Israel^4^ | 2009 |
| v226 (2322) | 1 | Fish | Israel^4^ | 1997 |
| v227 (101/4) | 1 | Fish | Israel^4^ | 1997 |
| v228 (2321) | 1 | Fish | Israel^4^ | 1997 |
| v225 (5/1) | 1 | Water | Israel^4^ | 1997 |
| yb10 | 1 | Fish | Israel | 2004 |
| yb12 | 1 | Fish | Israel | 2004 |
| yb13 | 1 | Fish | Israel | 2004 |
| yb14 | 1 | Fish | Israel | 2004 |
| yb15 | 1 | Fish | Israel | 2004 |
| yb17 | 1 | Fish | Israel | 2004 |
| yb18 | 1 | Fish | Israel | 2004 |
| yb2 | 1 | Fish | Israel | 2004 |
| yb20 | 1 | Fish | Israel | 2004 |
| yb21 | 1 | Fish | Israel | 2004 |
| yb22 | 1 | Fish | Israel | 2004 |
| yb23 | 1 | Fish | Israel | 2004 |
| yb24 | 1 | Fish | Israel | 2004 |
| yb25 | 1 | Fish | Israel | 2004 |
| yb4 | 1 | Fish | Israel | 2004 |
| yb44 | 1 | Fish | Israel | 2004 |
| yb47 | 1 | Fish | Israel | 2004 |
| yb48 | 1 | Fish | Israel | 2004 |
| yb5 | 1 | Fish | Israel | 2004 |
| yb50 | 1 | Fish | Israel | 2004 |
| yb54 | 1 | Fish | Israel | 2004 |
| yb55 | 1 | Fish | Israel | 2004 |
| yb56 | 1 | Fish | Israel | 2004 |
| yb57 | 1 | Fish | Israel | 2004 |
| yb6 | 1 | Fish | Israel | 2004 |
| yb7 | 1 | Fish | Israel | 2004 |
| yb8 | 1 | Fish | Israel | 2004 |
| yb9 | 1 | Fish | Israel | 2004 |
| yb110 | 1 | Fish | Israel | 2005 |
| yb162 | 1 | Fish | Israel | 2005 |
| yb163 | 1 | Fish | Israel | 2005 |
| yb164 | 1 | Fish | Israel | 2005 |
| yb167 | 1 | Fish | Israel | 2005 |
| yb172 | 1 | Fish | Israel | 2005 |
| yb175 | 1 | Fish | Israel | 2005 |
| yb176 | 1 | Fish | Israel | 2005 |
| yb177 | 1 | Fish | Israel | 2005 |
| yb178 | 1 | Fish | Israel | 2005 |
| yb179 | 1 | Fish | Israel | 2005 |
| yb180 | 1 | Fish | Israel | 2005 |
| yb181 | 1 | Fish | Israel | 2005 |
| yb182 | 1 | Fish | Israel | 2005 |
| yb183 | 1 | Fish | Israel | 2005 |
| yb185 | 1 | Fish | Israel | 2005 |
| yb187 | 1 | Fish | Israel | 2005 |
| yb189 | 1 | Fish | Israel | 2005 |
| yb190 | 1 | Fish | Israel | 2005 |
| yb191 | 1 | Fish | Israel | 2005 |
| yb192 | 1 | Fish | Israel | 2005 |
| yb193 | 1 | Fish | Israel | 2005 |
| yb62 | 1 | Fish | Israel | 2005 |
| yb64 | 1 | Fish | Israel | 2005 |
| yb70 | 1 | Fish | Israel | 2005 |
| yb74 | 1 | Fish | Israel | 2005 |
| yb78 | 1 | Fish | Israel | 2005 |
| yb79 | 1 | Fish | Israel | 2005 |
| yb81 | 1 | Fish | Israel | 2005 |
| yb82 | 1 | Fish | Israel | 2005 |
| yb85 | 1 | Fish | Israel | 2005 |
| yb134 | 1 | Fish | Israel | 2006 |
| yb135 | 1 | Fish | Israel | 2006 |
| yb136 | 1 | Fish | Israel | 2006 |
| yb195 | 1 | Fish | Israel | 2006 |
| yb196 | 1 | Fish | Israel | 2006 |
| yb197 | 1 | Fish | Israel | 2006 |
| yb198 | 1 | Fish | Israel | 2006 |
| yb199 | 1 | Fish | Israel | 2006 |
| yb200 | 1 | Fish | Israel | 2006 |
| yb202 | 1 | Fish | Israel | 2006 |
| yb203 | 1 | Fish | Israel | 2006 |
| yb204 | 1 | Fish | Israel | 2006 |
| yb205 | 1 | Fish | Israel | 2006 |
| yb206 | 1 | Fish | Israel | 2006 |
| yb207 | 1 | Fish | Israel | 2006 |
| yb208 | 1 | Fish | Israel | 2006 |
| yb210 | 1 | Fish | Israel | 2006 |
| yb211 | 1 | Fish | Israel | 2006 |
| yb214 | 1 | Fish | Israel | 2006 |
| yb215 | 1 | Fish | Israel | 2006 |
| yb216 | 1 | Fish | Israel | 2006 |
| yb218 | 1 | Fish | Israel | 2006 |
| yb219 | 1 | Fish | Israel | 2006 |
| yb221 | 1 | Fish | Israel | 2006 |
| 3012 | 1 | Fish | Israel | 2008 |
| 3032 | 1 | Fish | Israel | 2008 |
| 3072 | 1 | Fish | Israel | 2008 |
| 3162 | 1 | Fish | Israel | 2008 |
| 3182 | 1 | Fish | Israel | 2008 |
| 3422 | 1 | Fish | Israel | 2008 |
| 3442 | 1 | Fish | Israel | 2008 |
| 3472 | 1 | Fish | Israel | 2008 |
| 3482 | 1 | Fish | Israel | 2008 |
| 3492 | 1 | Fish | Israel | 2008 |
| 3512 | 1 | Fish | Israel | 2008 |
| 3542 | 1 | Fish | Israel | 2008 |
| 3572 | 1 | Fish | Israel | 2008 |
| 3612 | 1 | Fish pond sediment | Israel | 2009 |
| L49 | 1 | Brackish water | Japan | 1988 |
| N87 | 1 | Clinical | Japan | 1987 |
| KH03 | 1 | Clinical | Japan | 2003 |
| YN03 | 1 | Clinical | Japan | 2003 |
| CS9133 | 1 | Clinical | South Korea | Unknown |
| 94385 | 1 | Clinical | Spain | 2001 |
| A2 | 1 | Diseased eel | Spain | 2000 |
| An4 | 1 | Diseased eel | Spain | 2000 |
| PD-1 | 1 | Eel tank water | Spain | 2001 |
| PD-2-66 | 1 | Eel tank water | Spain | 2003 |
| Riu-1 | 1 | Seawater | Spain | 2003 |
| Riu-3 | 1 | Seawater | Spain | 2003 |
| 534 | 1 | Diseased eel | Sweden | Unknown |
| CG106 | 1 | Oyster | Taiwan | 1993 |
| CG111 | 1 | Seawater | Taiwan | 1993 |
| MLT 362 | 1 | Oyster | United States | 1991 |
| JE | 1 | Oyster | United States | Unknown |
| vv 425 | 1 | Oyster | United States | Unknown |
| MLT 406 | 1 | Seawater | United States | 1991 |
| CECT 5165 | 1 | Seawater | United States | Unknown |
| MLT404 | 1 | Seawater | United States | Unknown |
| vv 352 | 1 | Seawater | United States | Unknown |
| CECT 4866 | 2 | Clinical | Australia | Unknown |
| 94-8-112 | 2 | Clinical | Denmark | 1994 |
| 90-2-11 | 2 | Diseased eel | Denmark | 1990 |
| 95-8-6 | 2 | Diseased eel | Denmark | 1995 |
| 960426-1/4C | 2 | Diseased eel | Denmark | 1996 |
| CECT 7029 | 2 | Diseased eel | Denmark | 2004 |
| 94-9-123 | 2 | Seawater | Denmark | 1994 |
| v207 (03:96.8.6) | 2 | Diseased eel | Denmark^4^ | 1996 |
| v208 (03:96.8.7) | 2 | Diseased eel | Denmark^4^ | 1996 |
| v209 (03/04:95.8.161) | 2 | Diseased eel | Denmark^4^ | 2004 |
| v210 (03/04:95.8.162) | 2 | Diseased eel | Denmark^4^ | 2004 |
| v211 (4:96.7.137) | 2 | Diseased eel | Denmark^4^ | 2004 |
| v212 (04:96.7.138) | 2 | Diseased eel | Denmark^4^ | 2004 |
| CIP 81.9 | 2 | Clinical | France | 1981 |
| CECT 4862 | 2 | Diseased eel | Japan | 1979 |
| CECT 898 | 2 | Diseased eel | Japan | 1979 |
| CECT 4868 | 2 | Diseased eel | Norway | 1990 |
| CECT 4601 | 2 | Diseased eel | Spain | 1989 |
| CECT 4602 | 2 | Diseased eel | Spain | 1990 |
| CECT 4607 | 2 | Diseased eel | Spain | 1992 |
| CECT 4864 | 2 | Diseased eel | Spain | 1994 |
| CECT 4998 | 2 | Diseased eel | Spain | 1997 |
| CECT 5139 | 2 | Diseased eel | Spain | 1998 |
| CECT 4999 | 2 | Diseased eel | Spain | 1999 |
| CECT 5198 | 2 | Diseased eel | Spain | 1999 |
| CECT 5343 | 2 | Diseased eel | Spain | 2000 |
| CECT 5768 | 2 | Diseased eel | Spain | 2001 |
| A10 | 2 | Diseased eel | Spain | 2002 |
| CECT 5689 | 2 | Diseased eel | Spain | 2002 |
| CECT 5763 | 2 | Eel tank water | Spain | 2002 |
| PD-2-47 | 2 | Eel tank water | Spain | 2003 |
| PD-2-52 | 2 | Eel tank water | Spain | 2003 |
| PD-2-55 | 2 | Eel tank water | Spain | 2003 |
| CECT 5762 | 2 | Healthy eel | Spain | 2002 |
| Riu-2 | 2 | Seawater | Spain | 2003 |
| CECT 4870 | 2 | Diseased eel | Sweden | 1991 |
| 536 | 2 | Diseased eel | Sweden | Unknown |
| UE516 | 2 | Diseased Japanese eel | Taiwan | Unknown |
| CECT 4865 | 2 | Diseased shrimp | Taiwan | Unknown |
| v213 (145/96) | 3 | Clinical | Israel^4^ | 1996 |
| v214 (58/97) | 3 | Clinical | Israel^4^ | 1997 |
| v215 (11028/97) | 3 | Clinical | Israel^4^ | 1997 |
| v216 (162/97) | 3 | Clinical | Israel^4^ | 1997 |
| v217 (1033/97) | 3 | Clinical | Israel^4^ | 1997 |
| v218 (1/98) | 3 | Clinical | Israel^4^ | 1998 |
| v219 (1/99) | 3 | Clinical | Israel^4^ | 1999 |
| v220 (5/00) | 3 | Clinical | Israel^4^ | 2000 |
| v221 (8/00) | 3 | Clinical | Israel^4^ | 2000 |
| v222 (1/01) | 3 | Clinical | Israel^4^ | 2001 |
| v223 (10/02) | 3 | Clinical | Israel^4^ | 2002 |
| v254 (8/03) | 3 | Clinical | Israel^4^ | 2003 |
| v247 (2/04) | 3 | Clinical | Israel^4^ | 2004 |
| v248 (3/04) | 3 | Clinical | Israel^4^ | 2004 |
| v249 (4/04) | 3 | Clinical | Israel^4^ | 2004 |
| v250 (5/04) | 3 | Clinical | Israel^4^ | 2004 |
| v251 (6/04) | 3 | Clinical | Israel^4^ | 2004 |
| v253 (9/04) | 3 | Clinical | Israel^4^ | 2004 |
| v236 (1/05) | 3 | Clinical | Israel^4^ | 2005 |
| v237 (2/05) | 3 | Clinical | Israel^4^ | 2005 |
| v238 (5/05) | 3 | Clinical | Israel^4^ | 2005 |
| v239 (6/05) | 3 | Clinical | Israel^4^ | 2005 |
| v240 (7/05) | 3 | Clinical | Israel^4^ | 2005 |
| v241 (8/05) | 3 | Clinical | Israel^4^ | 2005 |
| v242 (9/05) | 3 | Clinical | Israel^4^ | 2005 |
| v243 (10/05) | 3 | Clinical | Israel^4^ | 2005 |
| v244 (11/05) | 3 | Clinical | Israel^4^ | 2005 |
| v245 (3/05) | 3 | Clinical | Israel^4^ | 2005 |
| v233 (1/06) | 3 | Clinical | Israel^4^ | 2006 |
| v234 (2/06) | 3 | Clinical | Israel^4^ | 2006 |
| v256 (2/09) | 3 | Clinical | Israel^4^ | 2009 |
| v257 (3/09) | 3 | Clinical | Israel^4^ | 2009 |
| yb1 | 3 | Fish | Israel | 2004 |
| yb101 | 3 | Fish | Israel | 2005 |
| yb108 | 3 | Fish | Israel | 2005 |
| yb109 | 3 | Fish | Israel | 2005 |
| yb114 | 3 | Fish | Israel | 2005 |
| yb115 | 3 | Fish | Israel | 2005 |
| yb116 | 3 | Fish | Israel | 2005 |
| yb117 | 3 | Fish | Israel | 2005 |
| yb118 | 3 | Fish | Israel | 2005 |
| yb63 | 3 | Fish | Israel | 2005 |
| yb66 | 3 | Fish | Israel | 2005 |
| yb71 | 3 | Fish | Israel | 2005 |
| yb72 | 3 | Fish | Israel | 2005 |
| yb73 | 3 | Fish | Israel | 2005 |
| yb87 | 3 | Fish | Israel | 2005 |
| yb88 | 3 | Fish | Israel | 2005 |
| yb89 | 3 | Fish | Israel | 2005 |
| yb91 | 3 | Fish | Israel | 2005 |
| yb93 | 3 | Fish | Israel | 2005 |
| yb95 | 3 | Fish | Israel | 2005 |
| yb127 | 3 | Fish | Israel | 2006 |
| yb128 | 3 | Fish | Israel | 2006 |
| yb129 | 3 | Fish | Israel | 2006 |
| yb131 | 3 | Fish | Israel | 2006 |
| yb132 | 3 | Fish | Israel | 2006 |
| yb137 | 3 | Fish | Israel | 2006 |
| yb140 | 3 | Fish | Israel | 2006 |
| yb141 | 3 | Fish | Israel | 2006 |
| yb144 | 3 | Fish | Israel | 2006 |
| yb146 | 3 | Fish | Israel | 2006 |
| yb156 | 3 | Fish | Israel | 2006 |
| 3042 | 3 | Fish | Israel | 2008 |
| 3282 | 3 | Fish | Israel | 2008 |
| v229 (105) | 3 | Fish | Israel^4^ | 2000 |
| v231 (12) | 3 | Fish | Israel^4^ | 2002 |
| v232 (8/03e) | 3 | Fish | Israel^4^ | 2003 |
| v224 (7/03) | ND | Clinical | Israel^4^ | 2003 |
| v246 (4/05) | ND | Clinical | Israel^4^ | 2005 |
| v235 (3/06) | ND | Clinical | Israel^4^ | 2006 |
| yb158 | ND | Fish | Israel | 2005 |
| yb160 | ND | Fish | Israel | 2005 |
| yb161 | ND | Fish | Israel | 2005 |
| yb165 | ND | Fish | Israel | 2005 |
| yb166 | ND | Fish | Israel | 2005 |
| yb171 | ND | Fish | Israel | 2005 |
| yb184 | ND | Fish | Israel | 2005 |
| yb186 | ND | Fish | Israel | 2005 |
| yb188 | ND | Fish | Israel | 2005 |
| yb209 | ND | Fish | Israel | 2006 |
| yb217 | ND | Fish | Israel | 2006 |

^1^ Isolation source was previously described for most strain [12,13,23] excluding the first 16 strains ^2^ that were isolated in this study from environmental fish samples in Israel during 2008.

^3^ ND= not determined - Unidentified biotype, all strains were clustered to clade A phylogroup [27].

^4^ Isolates are part of The Israeli Ministry of Health (IMH) collection. CDC, Centers for Disease Control and Prevention.
