# Supplementary material for: Genome-Wide SNP-Genotyping Array to Study the Evolution of the Human Pathogen Vibrio vulnificus Biotype 3
Source: PLoS One. 2014 Dec 19;9(12):e114576. doi: 10.1371/journal.pone.0114576 (PMC4272304; doi:10.1371/journal.pone.0114576)
Supplement: S2 Table — Targeted genes used to mine SNPs in biotype 3 and biotype 1 genomes. (DOCX) [file pone.0114576.s005.docx]

Table S2: Targeted genes used to mine SNPs in biotype 3 and biotype 1 genomes.

| **Locus** | **Product** | **Gene function/source** |
| --- | --- | --- |
| VV0220^1^ | type II secretory pathway, component EpsJ | Virulence factor |
| VV0341^1^ | UDP-N-acetylglucosamine 2-epimerase | Virulence factor |
| VV0971^1^ | flagellin | Virulence factor |
| VV1491^1^ | type IV pilus (Tfp) assembly protein PilF | Virulence factor |
| VV2792^1^ | S-ribosylhomocysteinase | Virulence factor |
| VVA0389^1^ | glycosyltransferase | Virulence factor |
| VVA0618^1^ | catechol siderophore ABC transporter, permease protein | Virulence factor |
| VVA0965^1^ | cytotoxin, cytolysin precursor VvhA | Virulence factor |
| VVA1032^1^ | RTX toxin activating protein | Virulence factor |
| VVA1300^1^ | vulnibactin-specific isochorismate synthase | Virulence factor |
| VVA1465^1^ | Zinc metalloprotease, vibriolysin | Virulence factor |
| VV3-258^2^ | IncF plasmid conjugative transfer pilus assembly protein TraE | Plasmid gene |
| VV3-267^2^ | IncF plasmid conjugative transfer pilus assembly protein TraF | Plasmid gene |
| VV3-269^2^ | IncF plasmid conjugative transfer pilus assembly protein TraH | Plasmid gene |
| VV3-824^2^ | hypothetical protein | Plasmid gene |
| VV3-499^2^ | hypothetical protein | Unique biotype 3 gene |
| VV3-503^2^ | hypothetical protein | Unique biotype 3 gene |
| VV3-1607^2^ | hypothetical protein | Unique biotype 3 gene |
| VV3-1916^2^ | hypothetical protein | Unique biotype 3 gene |
| VV3-1920^2^ | putative conjugative transfer protein TraD | Unique biotype 3 gene |
| VV3-2848^2^ | DegT/DnrJ/EryC1/StrS aminotransferase | Unique biotype 3 gene |
| VV3-2850^2^ | hypothetical protein | Unique biotype 3 gene |
| VV3-3339^2^ | RTX toxins and related Ca2+-binding protein | Unique biotype 3 gene |
| VV3-3415^2^ | hypothetical protein | Unique biotype 3 gene |

^1^ Based on *V. vulnificus* strain YJ016 [53].

^2^ Based on *V. vulnificus* strain VVyb1(BT3) [55].
