# Supplementary material for: Genome-Wide SNP-Genotyping Array to Study the Evolution of the Human Pathogen Vibrio vulnificus Biotype 3
Source: PLoS One. 2014 Dec 19;9(12):e114576. doi: 10.1371/journal.pone.0114576 (PMC4272304; doi:10.1371/journal.pone.0114576)
Supplement: S3 Table — PCR primers for sequence analysis of 30 strains performed for SNP discovery. (DOCX) [file pone.0114576.s006.docx]

Table S3: PCR primers for sequence analysis of 30 strains performed for SNP discovery.

| Locus | Forward primer (5'-3') |  | Reverse primer (5'-3') | Tm (^o^C) |
| --- | --- | --- | --- | --- |
| VV1068^1^ | TTAGGTGCTTGGTTGAACT |  | CGCCGATTACTTTCAGTTA | 55 |
| VV2310^1^ | CGCAAGATTTCGGACAATGAT |  | ATGGAAAGGCACTGATTATGG | 55 |
| VVA0555^1^ | TGAAGTCTCAACACTAGAGGA |  | TCTTTATAGAGTGGTACTGCC | 55 |
| VV3-0414^2^ | GTGTTAAGTCGGTTACAGGTT |  | AGCACAAGGTAGTACATCA | 55 |
| VV3-0415^2^ | TTAGCACACACACATCAGCAA |  | TCATAGGTGTCATCCAACTCT | 55 |
| VV3-1164^2^ | ACACCGTTAGAGGACTATATC |  | CACAAGCCAGTTGAAACACTA | 55 |
| VV3-1917^2^ | TGATGGAGGTTGGTAATGACT |  | AGTCTATTTGGTGGATCGGTA | 55 |

^1^ Based on *V. vulnificus* strain YJ016 [53].

^2^ Based on *V. vulnificus* strain VVyb1(BT3) [55].
