# Supplementary material for: Genome-Wide SNP-Genotyping Array to Study the Evolution of the Human Pathogen Vibrio vulnificus Biotype 3
Source: PLoS One. 2014 Dec 19;9(12):e114576. doi: 10.1371/journal.pone.0114576 (PMC4272304; doi:10.1371/journal.pone.0114576)
Supplement: S4 Table — The 574 SNP loci and alleles used in this study. (DOCX) [file pone.0114576.s007.docx]

Table S4: The 574 SNP loci and alleles used in this study.

| **SNP locus** | **Allele 1** | **Allele 2** |
| --- | --- | --- |
| VV0003a | T | A |
| VV0005 | G | A |
| VV0011b | G | T |
| VV0012 | C | T |
| VV0013a | C | T |
| VV0014h^#^ | G | A |
| VV0028a | A | G |
| VV0035d | A | T |
| VV0043 | A | C |
| VV0044c | T | C |
| VV0048_324* | T | A |
| VV0048_438*^,#^ | C | T |
| VV0059 | G | A |
| VV0062 | C | A |
| VV0065d | T | G |
| VV0081b | A | C |
| VV0083e | G | A |
| VV0087b | G | C |
| VV0100b | G | A |
| VV0119 | G | T |
| VV0121 | G | A |
| VV0122a | A | G |
| VV0123a | G | A |
| VV0168b | T | C |
| VV0177a | C | T |
| VV0178_100*^,#^ | G | A |
| VV0178_211*^,#^ | T | C |
| VV0178_326* | A | C |
| VV0178_572* | T | A |
| VV0179b | A | C |
| VV0187f | C | T |
| VV0204b | T | G |
| VV0207a | T | C |
| VV0208c | G | A |
| VV0220_186 | C | T |
| VV0220_405 | T | C |
| VV0236a | A | G |
| VV0239 | A | C |
| VV0242 | A | G |
| VV0282 | A | G |
| VV0285c | G | A |
| VV0297b | A | G |
| VV0324 | A | G |
| VV0341_508 | T | G |
| VV0341_654 | G | C |
| VV0374 | C | T |
| VV0375b | T | C |
| VV0378 | G | A |
| VV0389 | G | T |
| VV0395 | C | T |
| VV0399b | A | T |
| VV0412b | T | A |
| VV0415_135*^,#^ | C | T |
| VV0415_297*^,#^ | T | C |
| VV0415_432*^,#^ | C | T |
| VV0418a | T | C |
| VV0419 | T | C |
| VV0427a | T | A |
| VV0436 | C | T |
| VV0438b | T | C |
| VV0448a | G | A |
| VV0452a | C | A |
| VV0461b | C | T |
| VV0464b | C | T |
| VV0465a | C | T |
| VV0467a^#^ | A | C |
| VV0471 | G | A |
| VV0472c | T | C |
| VV0478c | C | A |
| VV0479a | A | T |
| VV0482a | C | T |
| VV0485b | C | A |
| VV0486c | G | C |
| VV0495b | A | G |
| VV0496a | A | C |
| VV0504c | T | C |
| VV0562d | G | A |
| VV0588c | A | T |
| VV0595a | A | G |
| VV0599 | T | C |
| VV0609a | C | T |
| VV0610f | G | A |
| VV0612a | G | C |
| VV0614 | T | A |
| VV0618d | C | T |
| VV0623c | A | C |
| VV0624c | T | A |
| VV0633b | C | A |
| VV0635 | G | T |
| VV0648 | C | T |
| VV0651d | G | A |
| VV0655 | T | C |
| VV0674c | A | T |
| VV0678c | A | G |
| VV0687c | T | G |
| VV0688a | T | C |
| VV0701g | A | G |
| VV0729d | G | T |
| VV0730c | G | A |
| VV0741e | G | T |
| VV0745a | G | A |
| VV0746a | C | T |
| VV0747 | A | G |
| VV0748a | A | T |
| VV0749 | G | A |
| VV0755a | C | T |
| VV0759a | C | T |
| VV0762a | G | A |
| VV0763b | A | G |
| VV0766 | C | T |
| VV0767 | A | G |
| VV0770a | A | C |
| VV0774b | C | A |
| VV0776d | C | G |
| VV0832a | C | T |
| VV0855b | C | T |
| VV0858a | C | T |
| VV0859a | A | T |
| VV0862 | C | T |
| VV0865c | T | C |
| VV0866a | C | A |
| VV0883a | T | C |
| VV0895c | C | T |
| VV0898d | A | T |
| VV0900c | T | C |
| VV0903 | A | G |
| VV0909b | C | T |
| VV0918b | A | G |
| VV0925b | C | T |
| VV0931b | G | A |
| VV0935a | C | A |
| VV0942a | T | C |
| VV0960 | C | T |
| VV0962 | A | C |
| VV0963 | G | A |
| VV0968a | A | G |
| VV0971_345 | G | A |
| VV0971_873 | G | A |
| VV0982e | T | C |
| VV0989c | G | A |
| VV1000b | A | G |
| VV1002a | T | C |
| VV1003 | T | C |
| VV1006c | A | G |
| VV1014 | A | G |
| VV1020b | T | C |
| VV1033a | G | A |
| VV1034a | A | G |
| VV1041j | C | T |
| VV1046 | G | T |
| VV1048c | T | C |
| VV1053b | T | C |
| VV1067 | A | C |
| VV1068_275* | T | G |
| VV1068_419* | G | A |
| VV1092c | C | T |
| VV1095 | T | C |
| VV1096b | A | G |
| VV1097a | C | A |
| VV1098d | C | T |
| VV1104 | G | A |
| VV1105b | C | T |
| VV1106 | G | A |
| VV1116b | C | T |
| VV1118b | G | T |
| VV1119a | G | T |
| VV1120e | T | C |
| VV1148 | A | G |
| VV1155a | C | A |
| VV1188 | C | T |
| VV1189a | G | T |
| VV1190c | G | A |
| VV1191a | G | A |
| VV1193d | C | A |
| VV1197_322*^,#^ | G | A |
| VV1197_61*^,#^ | G | C |
| VV1200b | T | G |
| VV1206c | A | C |
| VV1213a | A | C |
| VV1216b | C | A |
| VV1218a | G | T |
| VV1219c | A | G |
| VV1220 | T | G |
| VV1221h | A | G |
| VV1232c | C | T |
| VV1234b | C | T |
| VV1243c | A | G |
| VV1261a | A | G |
| VV1272a | T | C |
| VV1278 | A | G |
| VV1283b | G | T |
| VV1299 | T | C |
| VV1300c | C | T |
| VV1304b | G | T |
| VV1328a | T | C |
| VV1350a | T | C |
| VV1352e | A | G |
| VV1353a | G | A |
| VV1354b | T | A |
| VV1355b | C | T |
| VV1360 | A | G |
| VV1368 | G | A |
| VV1381b | A | G |
| VV1406c | A | G |
| VV1407a | G | A |
| VV1410 | G | A |
| VV1420a | T | C |
| VV1463a | T | C |
| VV1475a | A | G |
| VV1480b | T | C |
| VV1483_168* | G | C |
| VV1483_372*^,#^ | G | A |
| VV1491_147 | C | A |
| VV1491_630 | T | C |
| VV1529e | A | C |
| VV1559a | T | C |
| VV1605a | G | A |
| VV1606 | C | T |
| VV1642d | A | T |
| VV1646b | T | A |
| VV1654d | C | T |
| VV1672b | G | A |
| VV16S_144 | G | A |
| VV16S_404 | C | T |
| VV16S_721 | A | G |
| VV16S_840 | C | T |
| VV1714b | T | C |
| VV1715 | G | T |
| VV1716 | T | C |
| VV1949c | G | C |
| VV1950 | C | T |
| VV1951b | C | T |
| VV1962a | A | G |
| VV1969c | C | G |
| VV1971 | C | T |
| VV1986b | T | C |
| VV1987a | G | T |
| VV2003 | T | A |
| VV2050 | T | C |
| VV2059b | T | G |
| VV2080a | C | A |
| VV2121a | T | C |
| VV2135d | A | T |
| VV2272a | A | C |
| VV2275 | T | C |
| VV2284 | C | T |
| VV2285 | A | G |
| VV2287b | T | C |
| VV2299b | T | C |
| VV2300d | T | C |
| VV2303a | A | G |
| VV2310_202 | A | G |
| VV2310_437 | C | T |
| VV2312g | G | A |
| VV2313c | T | C |
| VV2314b | A | G |
| VV2337b | C | T |
| VV2339c | C | T |
| VV2353b | T | C |
| VV2355b | G | A |
| VV2404b | T | G |
| VV2411a | A | C |
| VV2425c | G | A |
| VV2428b | A | G |
| VV2477a | C | T |
| VV2478 | C | T |
| VV2479c | T | G |
| VV2481b | C | T |
| VV2482 | A | G |
| VV2487 | A | C |
| VV2490 | C | T |
| VV2494 | C | T |
| VV2500f | A | T |
| VV2541e | T | G |
| VV2544 | C | T |
| VV2545 | G | T |
| VV2546b | G | A |
| VV2547a | A | G |
| VV2551a | T | C |
| VV2555b | T | G |
| VV2556b | A | G |
| VV2559d | G | A |
| VV2572b | G | T |
| VV2580a | G | A |
| VV2581a | T | C |
| VV2585a | G | A |
| VV2588 | G | A |
| VV2589a | T | A |
| VV2590a | C | T |
| VV2602 | G | A |
| VV2603a | C | A |
| VV2614e | T | C |
| VV2624d | C | T |
| VV2637 | C | T |
| VV2648a | G | A |
| VV2678a | A | G |
| VV2683c | T | C |
| VV2687 | C | T |
| VV2706 | C | G |
| VV2707 | T | G |
| VV2708k | C | T |
| VV2709c | A | T |
| VV2715c | T | C |
| VV2723a | A | G |
| VV2745g | T | C |
| VV2746 | G | T |
| VV2759 | T | C |
| VV2763 | C | T |
| VV2774b | G | A |
| VV2786 | T | C |
| VV2792_384 | C | T |
| VV2792_90 | T | C |
| VV2794b | T | C |
| VV2799a | A | T |
| VV2800 | T | A |
| VV2803f | G | A |
| VV2804 | T | C |
| VV2805d | A | G |
| VV2812b | T | C |
| VV2813 | C | T |
| VV2814 | C | T |
| VV2818b | A | T |
| VV2819c | C | T |
| VV2827a | T | C |
| VV2830a | G | A |
| VV2833a | T | G |
| VV2836 | C | T |
| VV2839d | A | G |
| VV2846a | T | G |
| VV2849b | G | A |
| VV2857d | A | T |
| VV2858b | G | A |
| VV2868c | T | G |
| VV2875b | G | A |
| VV2879 | C | A |
| VV2898b | G | A |
| VV2907c | C | T |
| VV2919a | A | C |
| VV2920b | T | C |
| VV2935a | A | G |
| VV2936a | A | G |
| VV2941 | A | G |
| VV2942 | G | A |
| VV2950c | A | C |
| VV2952a | T | C |
| VV2956h | C | T |
| VV2959a | C | T |
| VV2960d | C | G |
| VV2965f | A | G |
| VV2975a | T | C |
| VV2978d | G | C |
| VV2985 | A | G |
| VV2988 | C | T |
| VV2999c | T | C |
| VV3000c | T | C |
| VV3004a | A | G |
| VV3007a | A | G |
| VV3009 | A | G |
| VV3015a | G | A |
| VV3017a | C | T |
| VV3029a | T | G |
| VV3031 | G | A |
| VV3043b | G | A |
| VV3054b | T | C |
| VV3058a | G | A |
| VV3072b | T | C |
| VV3074d | A | G |
| VV3079a | T | C |
| VV3087a | G | A |
| VV3098b | T | C |
| VV3111b | C | T |
| VV3114b | C | T |
| VV3-1164_121 | A | G |
| VV3-1164_240 | C | T |
| VV3-1164_421 | T | C |
| VV3-1164_540 | C | T |
| VV3119a | G | A |
| VV3120a | C | T |
| VV3133a | C | T |
| VV3134a | T | C |
| VV3137a | T | C |
| VV3139 | T | C |
| VV3141c | A | G |
| VV3159g | T | C |
| VV3-1607_121 | G | T |
| VV3-1607_240 | T | C |
| VV3162 | T | C |
| VV3163a | C | T |
| VV3164 | A | G |
| VV3172d | C | T |
| VV3177c | C | T |
| VV3179a | G | A |
| VV3181a | G | A |
| VV3183c | T | C |
| VV3187b | G | C |
| VV3-1916_121 | C | T |
| VV3-1916_240 | T | G |
| VV3-1916_660 | A | G |
| VV3-1917_121 | A | G |
| VV3-1917_240 | A | T |
| VV3-1917_541 | C | T |
| VV3-1917_660 | A | C |
| VV3-1920_301 | G | T |
| VV3-1920_420 | A | C |
| VV3202 | T | C |
| VV3203b | T | G |
| VV3206b | A | C |
| VV3207b | C | T |
| VV3208b | T | A |
| VV3215 | G | A |
| VV3223b | T | A |
| VV3226b | A | G |
| VV3228c | T | C |
| VV3234g | A | C |
| VV3239b | T | G |
| VV3242 | T | C |
| VV3243 | G | A |
| VV3254 | T | C |
| VV3-258_301 | G | A |
| VV3258a | T | C |
| VV3260b | T | C |
| VV3261a | C | A |
| VV3-267_601 | A | G |
| VV3-269_721 | G | A |
| VV3-2848_161 | C | T |
| VV3-2848_511 | A | T |
| VV3-2850_121 | T | G |
| VV3-2850_240 | T | C |
| VV3-2850_421 | C | T |
| VV3-3339_1081 | T | G |
| VV3-3339_1200 | G | A |
| VV3-3339_241 | G | T |
| VV3-3339_360 | T | C |
| VV3-3415_121 | C | T |
| VV3-3415_421 | C | T |
| VV3-3415_540 | A | G |
| VV3-414_156 | T | C |
| VV3-414_537 | C | T |
| VV3-415_605 | A | G |
| VV3-415_776 | T | C |
| VV3-499_148 | C | T |
| VV3-499_481 | G | A |
| VV3-499_600 | T | G |
| VV3-503_121 | A | G |
| VV3-503_361 | G | A |
| VV3-824_61 | C | A |
| VVA0003b | T | C |
| VVA0006 | A | G |
| VVA0028 | G | A |
| VVA0031c | A | G |
| VVA0040b | T | A |
| VVA0056b | C | T |
| VVA0067c | A | G |
| VVA0076h | C | T |
| VVA0078e | A | T |
| VVA0079a | A | T |
| VVA0081b | C | T |
| VVA0126a | G | A |
| VVA0161 | C | T |
| VVA0180c | A | G |
| VVA0181a | C | T |
| VVA0182a | C | T |
| VVA0203a | T | C |
| VVA0229 | A | G |
| VVA0267d | T | C |
| VVA0304c | C | T |
| VVA0370 | G | A |
| VVA0372 | C | T |
| VVA0375c | T | A |
| VVA0385c | C | T |
| VVA0399b | A | G |
| VVA0410b | T | C |
| VVA0411b | C | T |
| VVA0419 | T | C |
| VVA0425b | T | A |
| VVA0459 | T | C |
| VVA0461a | A | G |
| VVA0478 | A | G |
| VVA0500b | G | A |
| VVA0506a | C | T |
| VVA0514i | A | G |
| VVA0520d | T | G |
| VVA0521a | G | A |
| VVA0555_316 | A | G |
| VVA0555_62 | G | A |
| VVA0570 | T | C |
| VVA0586d | C | T |
| VVA0597a | C | T |
| VVA0598a | C | A |
| VVA0599a | G | A |
| VVA0630 | G | A |
| VVA0656d | G | T |
| VVA0658 | G | A |
| VVA0687 | A | G |
| VVA0691h | G | A |
| VVA0692 | G | A |
| VVA0713 | G | A |
| VVA0721 | G | A |
| VVA0754 | C | A |
| VVA0755c | G | T |
| VVA0785a | A | G |
| VVA0812a^#^ | A | T |
| VVA0821c | G | T |
| VVA0822 | T | C |
| VVA0823 | A | C |
| VVA0895a | C | T |
| VVA0906 | C | T |
| VVA0914c | T | C |
| VVA0926a | G | C |
| VVA0927a | G | A |
| VVA0965_321 | A | T |
| VVA0965_909 | T | A |
| VVA0971c | T | C |
| VVA0980c | G | A |
| VVA1007 | G | A |
| VVA1029b | T | A |
| VVA1032_265 | T | C |
| VVA1081 | T | G |
| VVA1093b | T | C |
| VVA1101a | A | G |
| VVA1106b | T | C |
| VVA1140a | C | T |
| VVA1200a | C | T |
| VVA1201a | C | T |
| VVA1202e | T | A |
| VVA1205a | T | C |
| VVA1206 | T | C |
| VVA1211b | C | A |
| VVA1232c | A | C |
| VVA1255 | A | C |
| VVA1261a | A | T |
| VVA1282a | T | C |
| VVA1299b | T | G |
| VVA1300_510 | C | T |
| VVA1300_978 | C | T |
| VVA1333b | C | T |
| VVA1349b | T | C |
| VVA1376c | G | A |
| VVA1405 | G | A |
| VVA1428c | T | C |
| VVA1465_1218 | C | T |
| VVA1465_1530 | T | C |
| VVA1476c | T | C |
| VVA1477b | C | T |
| VVA1509d | C | T |
| VVA1588c | A | C |
| VVA1601a | C | T |
| VVA1606a | G | A |
| VVA1608 | A | C |
| VVA1624 | C | A |
| VVA1648a | A | G |
| VVA1659a | A | G |
| VVA389_144 | T | C |
| VVA389_855 | C | T |
| VVA618_588 | G | T |
| VVA618_676 | G | T |
| VVglp_114* | C | T |
| VVglp_237* | T | C |
| VVglp_333*^,#^ | T | C |
| VVglp_423*^,#^ | ] | A |
| VVgyrB_225*^,#^ | T | C |
| VVgyrB_360* | T | C |
| VVgyrB_66*^,#^ | T | C |
| VVlysA_157*^,#^ | T | C |
| VVlysA_328* | C | T |
| VVmdh_316*^,#^ | A | G |
| VVmdh_62* | G | T |
| VVpntA_144*^,#^ | G | T |
| VVpntA_318*^,#^ | T | G |
| VVpyrC_102*^,#^ | A | T |
| VVpyrC_204*^,#^ | C | G |
| VVpyrC_315*^,#^ | A | C |
| VVtnaA_247*^,#^ | C | T |
| VVtnaA_67*^,#^ | G | A |

* SNPs used as array quality control

^#^ SNPs from 7 housekeeping (16 SNPS) and 5 conserved hypothetical genes (9 SNPs) that were used to predict the group origin of biotype 3 haplotype.


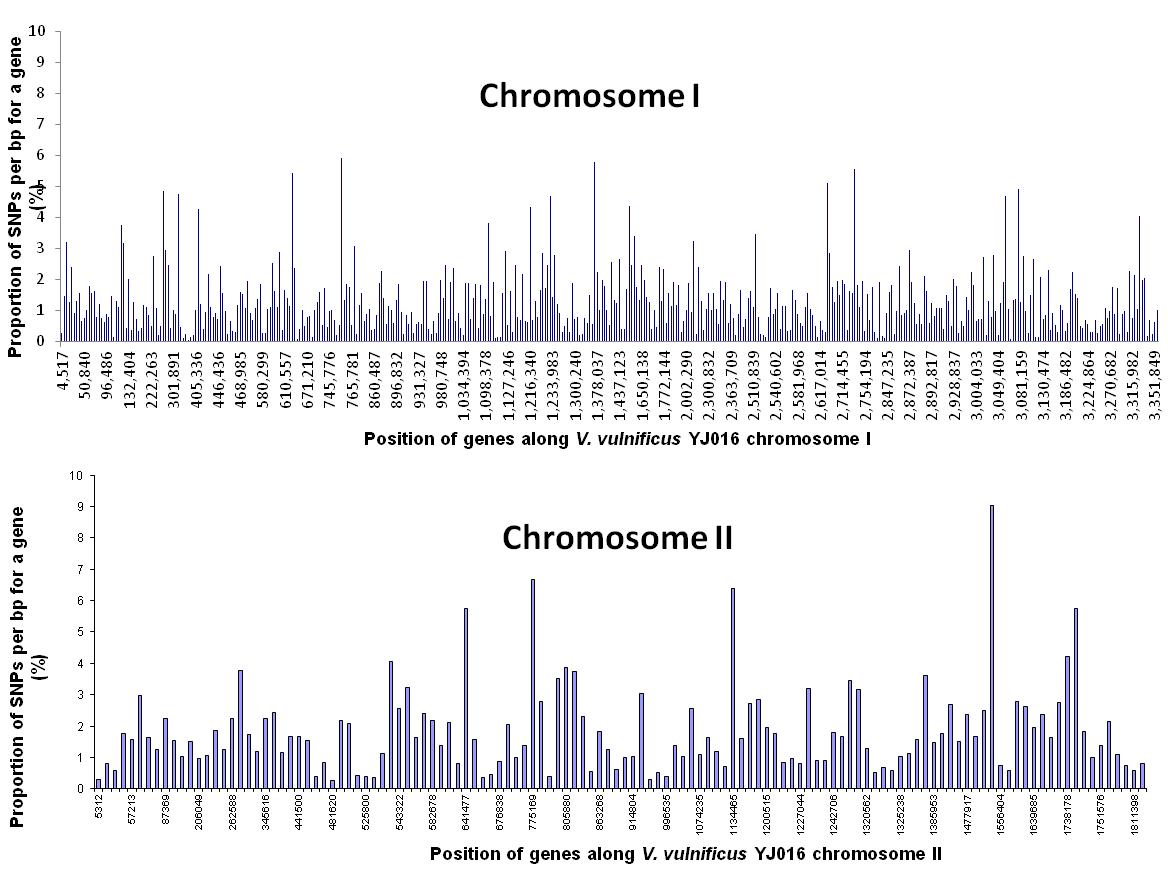


**Figure S1: Distribution of SNPs found between CMCP6 and YJ016 genomes presented along the *V. vulnificus* YJ016 genome.**


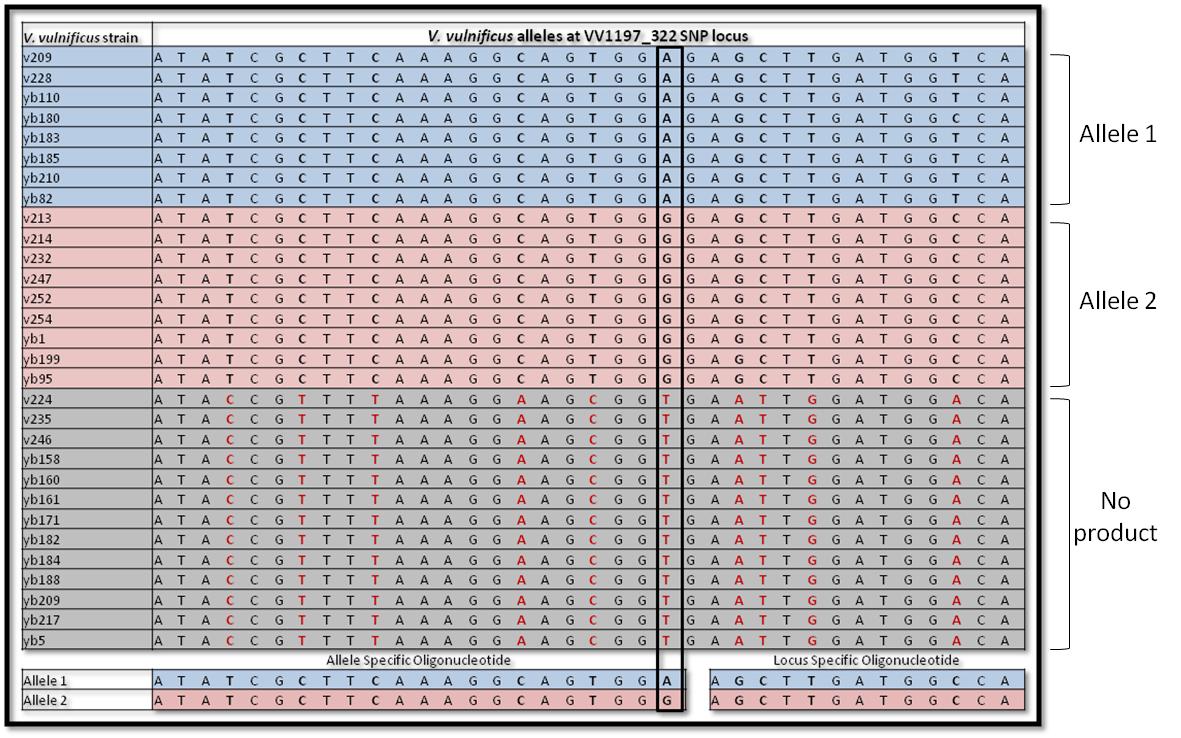


**Figure S2: Allelic variation among 30 *V. vulnificus* strains at SNP locus VV1197_322.**

**
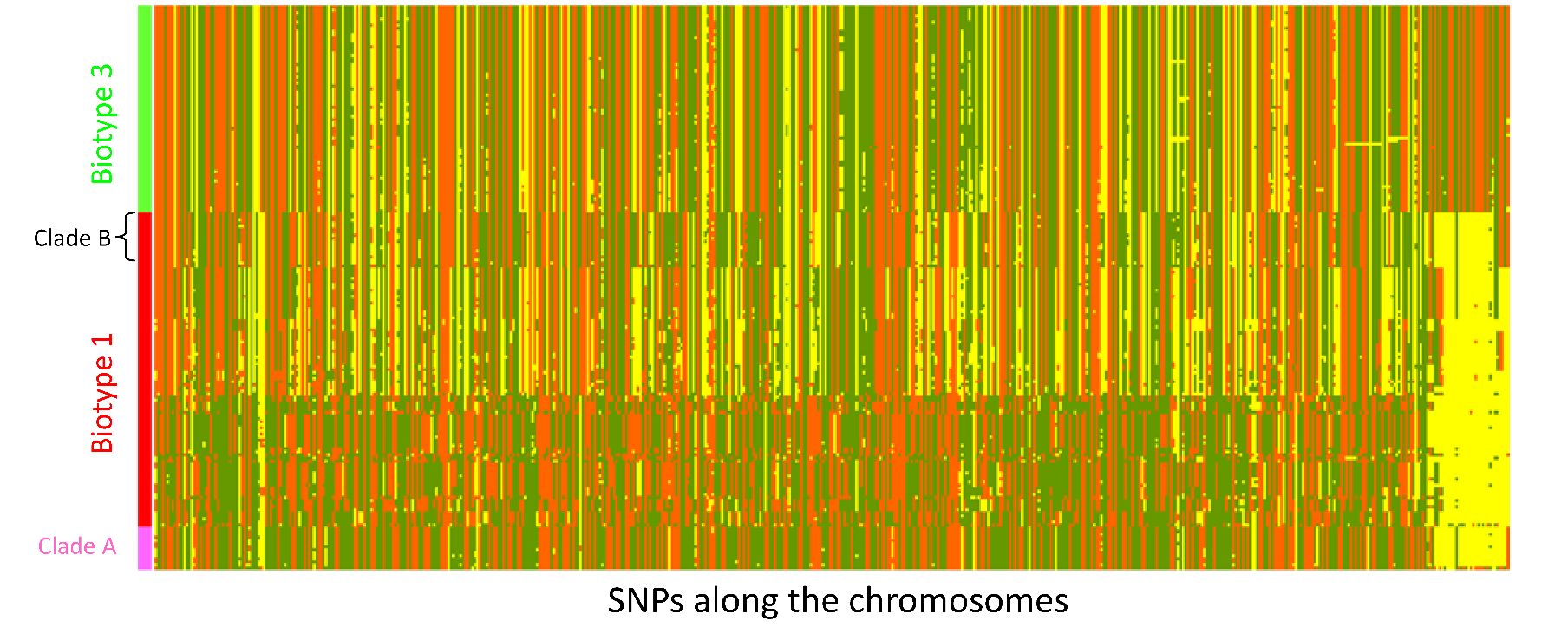
**

**Figure S3: Heat map generated for the GoldenGate data at 570 SNPs distributed along the bacterial chromosomes of 185 *V. vulnificus* strains isolated in Israel between 1996 and 2009.**
